# The intricate dance of RNA-binding proteins: unveiling the mechanisms behind male infertility

**DOI:** 10.1093/humupd/dmaf023

**Published:** 2025-08-22

**Authors:** Ying Gao, Yikun Zhou, Zhidan Hong, Binyu Ma, Xiaojie Wang, Linhang Nie, Ling Ma, Yuanzhen Zhang, Ming Zhang, Mei Wang

**Affiliations:** Center for Reproductive Medicine, Zhongnan Hospital of Wuhan University, Wuhan, Hubei, P.R. China; Clinical Medicine Research Center of Prenatal Diagnosis and Birth Health in Hubei Province, Wuhan, Hubei, P.R. China; Wuhan Clinical Research Center for Reproductive Science and Birth Health, Wuhan, Hubei, P.R. China; State Key Laboratory of Oral and Maxillofacial Reconstruction and Regeneration, Department of Orthodontics, School and Hospital of Stomatology, Wuhan University, Wuhan, Hubei, P.R. China; Center for Reproductive Medicine, Zhongnan Hospital of Wuhan University, Wuhan, Hubei, P.R. China; Clinical Medicine Research Center of Prenatal Diagnosis and Birth Health in Hubei Province, Wuhan, Hubei, P.R. China; Wuhan Clinical Research Center for Reproductive Science and Birth Health, Wuhan, Hubei, P.R. China; Center for Reproductive Medicine, Zhongnan Hospital of Wuhan University, Wuhan, Hubei, P.R. China; Center for Reproductive Medicine, Zhongnan Hospital of Wuhan University, Wuhan, Hubei, P.R. China; Center for Reproductive Medicine, Zhongnan Hospital of Wuhan University, Wuhan, Hubei, P.R. China; Center for Reproductive Medicine, Zhongnan Hospital of Wuhan University, Wuhan, Hubei, P.R. China; Center for Reproductive Medicine, Zhongnan Hospital of Wuhan University, Wuhan, Hubei, P.R. China; Clinical Medicine Research Center of Prenatal Diagnosis and Birth Health in Hubei Province, Wuhan, Hubei, P.R. China; Wuhan Clinical Research Center for Reproductive Science and Birth Health, Wuhan, Hubei, P.R. China; Center for Reproductive Medicine, Zhongnan Hospital of Wuhan University, Wuhan, Hubei, P.R. China; Clinical Medicine Research Center of Prenatal Diagnosis and Birth Health in Hubei Province, Wuhan, Hubei, P.R. China; Wuhan Clinical Research Center for Reproductive Science and Birth Health, Wuhan, Hubei, P.R. China; Center for Reproductive Medicine, Zhongnan Hospital of Wuhan University, Wuhan, Hubei, P.R. China; Clinical Medicine Research Center of Prenatal Diagnosis and Birth Health in Hubei Province, Wuhan, Hubei, P.R. China; Wuhan Clinical Research Center for Reproductive Science and Birth Health, Wuhan, Hubei, P.R. China

**Keywords:** RNA-binding protein, spermatogenesis, male infertility, piRNA, alternative splicing, transcription-translation decoupling

## Abstract

**BACKGROUND:**

RNA-binding proteins (RBPs) are indispensable for transcriptional and post-transcriptional processes during spermatogenesis, orchestrating germ cell proliferation, differentiation, and maturation. Despite their established importance, the contributions of RBPs in male infertility remain underexplored. Recently, a seminal *Science* publication reported an RBP atlas of 1744 murine testicular RBPs, 22 loss-of-function variants, and 137 deleterious missense variants identified in 1046 infertile patients, providing unprecedented opportunities to investigate their molecular and clinical relevance. Variants in RBP-related genes associated with azoospermia, oligozoospermia, teratozoospermia, and asthenozoospermia highlight their potential as diagnostic biomarkers and therapeutic targets. However, comprehensive analyses that integrate genetic, functional, and clinical insights are still lacking.

**OBJECTIVE AND RATIONALE:**

This review aims to systematically analyze the roles of RBPs in male infertility. Leveraging state-of-the-art datasets and experimental insights, it examines pathogenic variants and variants of uncertain significance (VUS), and elucidates the gene–disease relationships (GDRs). Furthermore, it explores known RBP functions across spermatogenesis stages and identifies candidate RBP genes. By integrating these findings, this work provides a comprehensive framework to advance the genetic understanding of RBPs, and their potential as clinical biomarkers and therapeutic targets in male infertility.

**SEARCH METHODS:**

We searched the PubMed database for articles until 13 July 2025, using the keywords ‘RNA-binding protein’, ‘male infertility’, ‘spermatogenesis’, ‘sperm’, ‘genetic variant’, ‘functional analyses’, and ‘knockout mouse model’. Pathogenic variants and VUS in 1744 RBP-coding genes, retrieved from the ClinVar and PubMed databases, were systematically analyzed to classify GDRs by the International Male Infertility Genomics Consortium database. Functional data from RBP knockout mouse models were assessed to elucidate stage-specific roles in spermatogenesis. Candidate RBP genes lacking knockout mouse models were identified by mining the RBP atlas, alongside data from the Genotype-Tissue Expression, Human Protein Atlas, and Uniprot databases. The clinical potential of RBPs as diagnostic biomarkers and therapeutic targets was also discussed.

**OUTCOMES:**

Our search generated ∼2000 records, and 331 relevant articles were ultimately included in the final text. Firstly, this review identified 177 pathogenic variants in 62 RBP genes and 91 VUS in 35 RBP genes, 15 of which have been confidently linked to human male infertility. Secondly, functional analyses of 124 RBP knockout mouse models revealed their stage-specific regulatory roles in spermatocytogenesis, spermatidogenesis, and spermiogenesis, offering insights into key processes such as piwi-interacting RNA biogenesis, chromatin remodeling, and RNA stability. Thirdly, 38 RBP genes lacking knockout mouse models were screened as candidate RBP genes in male infertility, underscoring their potential for future functional investigations. Finally, this review discusses the clinical potential of RBPs as biomarkers and therapeutic targets, including RNA-based drugs, small molecules, and gene editing technologies as innovative strategies to address RBP-related male infertility.

**WIDER IMPLICATIONS:**

This review highlights the role of RBPs in male infertility and offers a framework for integrating genetic, functional, and clinical data. By identifying candidate RBPs and their therapeutic potential, it lays the groundwork for future diagnostic advancements and personalized treatments in reproductive medicine.

**REGISTRATION NUMBER:**

N/A.

## Graphical abstract

**Figure:**
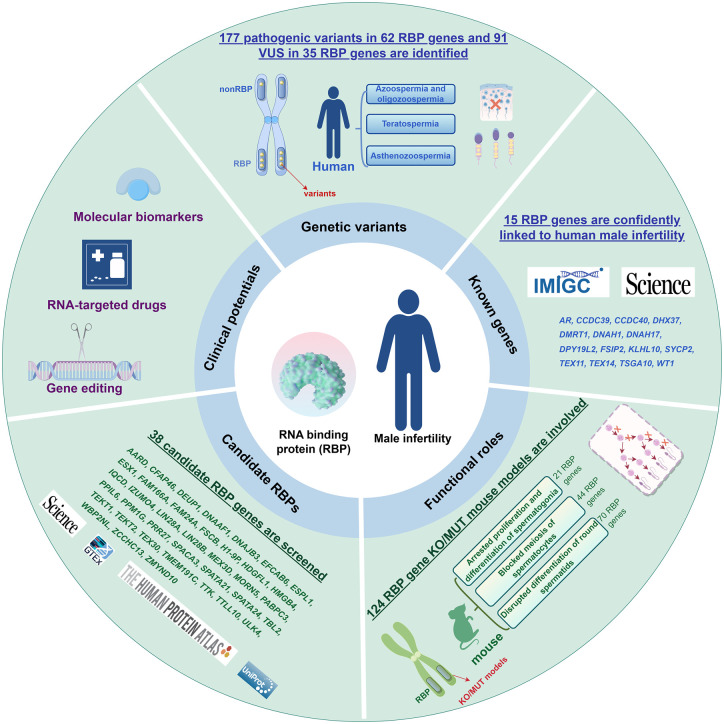
**Unraveling the genetic, functional, and clinical landscape of RNA-binding proteins in male infertility.** KO, knockout; MUT, mutant; RBP, RNA-binding protein; VUS, variants of uncertain significance. This image was created using FigDraw (www.figdraw.com: PWSAW89088).

## Introduction

Male infertility represents a critical global health challenge, affecting ∼5–10% of the male population and contributing to nearly 50% of infertility cases in couples (Agarwal *et al.*, 2021; Eisenberg *et al.*, 2023; Lillepea *et al.*, 2024). Despite significant advancements in diagnostic methodologies and assisted reproductive technologies, the molecular underpinnings of male infertility remain poorly characterized. Among the factors implicated in male reproductive health, RNA-binding proteins (RBPs) have emerged as pivotal regulators of spermatogenesis and sperm function, orchestrating intricate transcriptional and post-transcriptional regulatory networks essential for male germ cell development (Gebauer *et al.*, 2021; Li *et al.*, 2024c).

RBPs are a class of proteins that bind RNA molecules, modulating their stability, localization, splicing, and translation (Hentze *et al.*, 2018). Their dynamic expression and spatial localization in testicular tissues underscore their critical roles in maintaining the precise molecular regulation for normal spermatogenesis. Perturbations in RBP function—resulting from genetic mutations, aberrant expression, or environmental stressors—can disrupt multiple stages of spermatogenesis, leading to various forms of male infertility, including azoospermia, oligozoospermia, teratozoospermia, and asthenozoospermia.

A recent *Science* publication identified an RBP atlas along spermatogenesis, by utilizing RNA interactome capture to mouse male germ cells. Furthermore, whole-exome sequencing of over 1000 infertile men revealed a prominent role of RBPs in the human genetic architecture of male infertility (Li *et al.*, 2024c). This landmark study not only expands the molecular understanding of RBPs in spermatogenesis but also provides a valuable resource for further exploration of their roles in male infertility.

To explore more RBP-associated genetic factors in human male infertility, we screened male infertility-associated variants in 1744 testicular RBP genes using the PubMed and ClinVar databases (Li *et al.*, 2024c). One hundred seventy-seven pathogenic variants in 62 RBP genes and 91 variants of uncertain significance (VUS) in 35 RBP genes were identified. Each gene was annotated with its gene–disease relationship (GDR) classification by International Male Infertility Genomics Consortium (IMIGC, http://www.imigc.org) (Oud *et al.*, 2019; Houston *et al.*, 2021; Jiao *et al.*, 2021), providing a mutational landscape of RBP genes potentially implicated in male infertility. Among these, 15 RBP genes—including *AR, CCDC39, CCDC40, DHX37, DMRT1, DNAH1, DNAH17, DPY19L2, FSIP2, KLHL10, SYCP2, TEX11, TEX14*, *TSGA10*, and *WT1*—are confidently linked (moderate, strong, or definitive) to human male infertility. Beyond these 15 RBPs with confident GDRs in male infertility, the remaining RBP gene variants represent potential causative genes for male infertility, which require further investigation by future researchers. The characterization of these candidates may reveal new genetic causes of spermatogenic failure and guide future diagnostic and therapeutic approaches.

To investigate the molecular functions of RBP genes in male infertility, we analyzed fertility phenotypes in knockout mice of 1744 RBP genes. Of these, 124 exhibit male infertility and 25 show male subfertility, further elucidating their stage-specific regulatory roles in spermatogenesis, such as piwi-interacting RNA (piRNA) biogenesis, chromatin remodeling, and RNA stability. Additionally, we innovatively screened 38 candidate RBPs lacking knockout mouse models by integrating data from multiple high-quality bioinformatic resources. These uncharacterized genes represent a promising pool for future investigations in male infertility.

In this review, we performed an overview of genetic variants, functional roles, and clinical potentials in RBP genes for male infertility. By systematically summarizing current evidence, we provided a mutational landscape of RBP genes, identified key RBP genes confidently linked to male infertility, elucidated stage-specific regulatory roles of RBPs in spermatogenesis, and proposed candidates for future research. Our goal is to provide a comprehensive and up-to-date resource to guide further studies, supporting the development of diagnostic and therapeutic strategies targeting RBP dysfunction for male infertility.

## Methods

**Figure 1.**
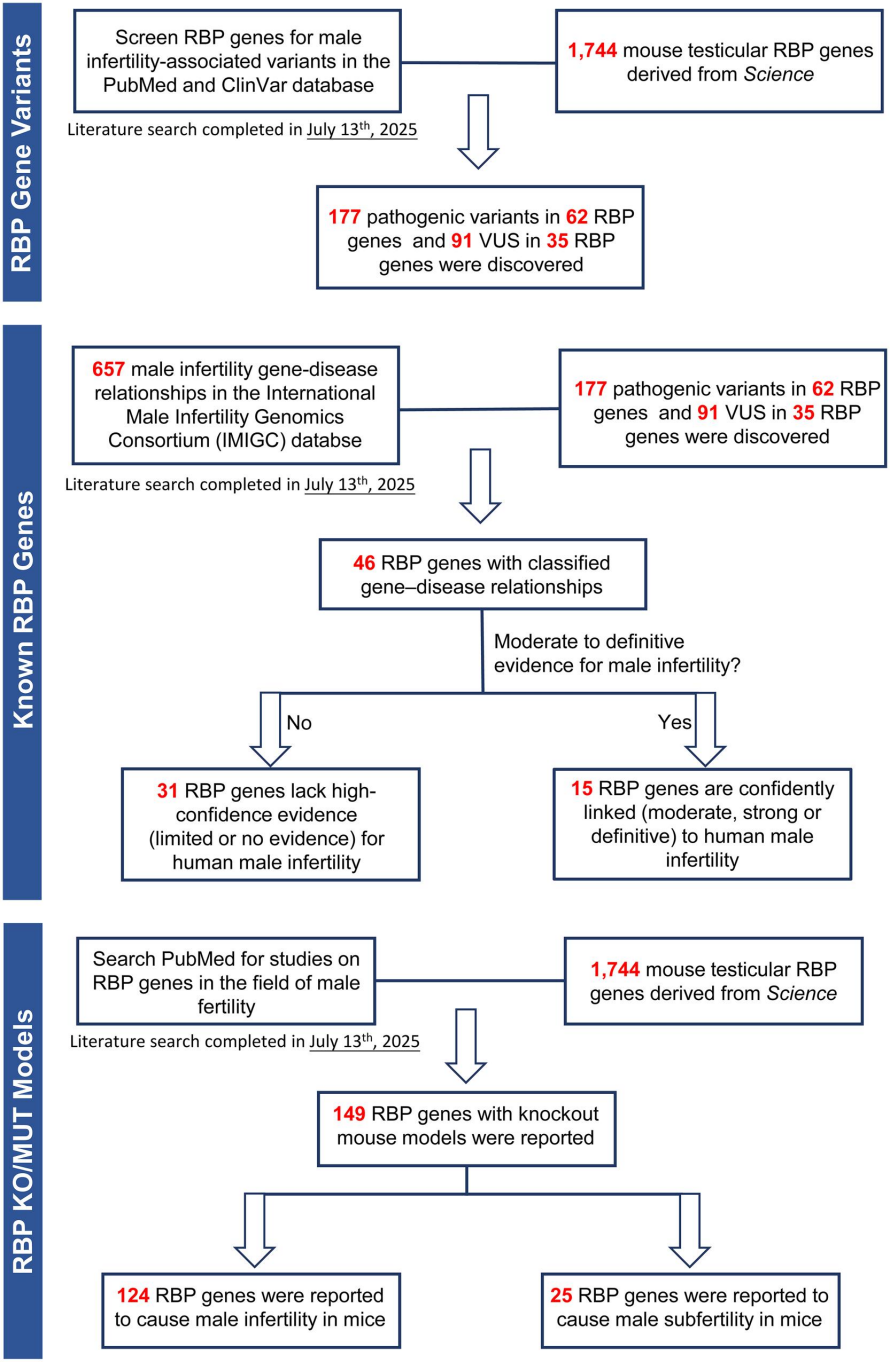
**Screening strategies for male infertility-related RNA-binding protein genes.** KO, knockout; MUT, mutant; RBP, RNA-binding protein; VUS, variants of uncertain significance.

We conducted a comprehensive literature search in PubMed for articles published up to 13 July 2025. The following keywords were used individually and in combination: ‘*RNA-binding protein*’, ‘*male infertility*’, ‘*spermatogenesis*’, ‘*sperm*’, ‘*genetic variant*’, ‘*functional analyses*’, and ‘*knockout mouse model*’. The aim was to identify studies investigating the genetic, functional, and clinical relevance of RBPs in male reproductive biology. Data on pathogenic variants and VUS in 1744 RBP-coding genes were retrieved from ClinVar and PubMed, and GDRs were systematically classified based on the criteria established by the IMIGC. Functional evidence from knockout mouse models was assessed to elucidate stage-specific roles of RBPs during spermatogenesis (Fig. 1). To identify candidate RBPs lacking knockout mouse models, we mined the RBP atlas and integrated transcriptomic and proteomic evidence from the Genotype-Tissue Expression (GTEx), Human Protein Atlas (HPA), and UniProt databases. We also examined the clinical potential of RBPs as diagnostic biomarkers and therapeutic targets for male infertility. Our search yielded ∼2000 records, of which 331 articles were selected based on relevance, methodological quality, and contribution to the scope of this review.

## Structure and biological functions of RBPs

RBPs are central to the intricate regulatory networks governing transcriptional and post-transcriptional gene expression. Their indispensable roles in RNA metabolism make them critical for processes such as spermatogenesis, where rapid and stage-specific changes in RNA stability, localization, splicing, and translation are required. This regulation ensures the precise control of gene expression programs essential for the development of functional spermatozoa.

### Structural characteristics of RBPs

**Figure 2.**
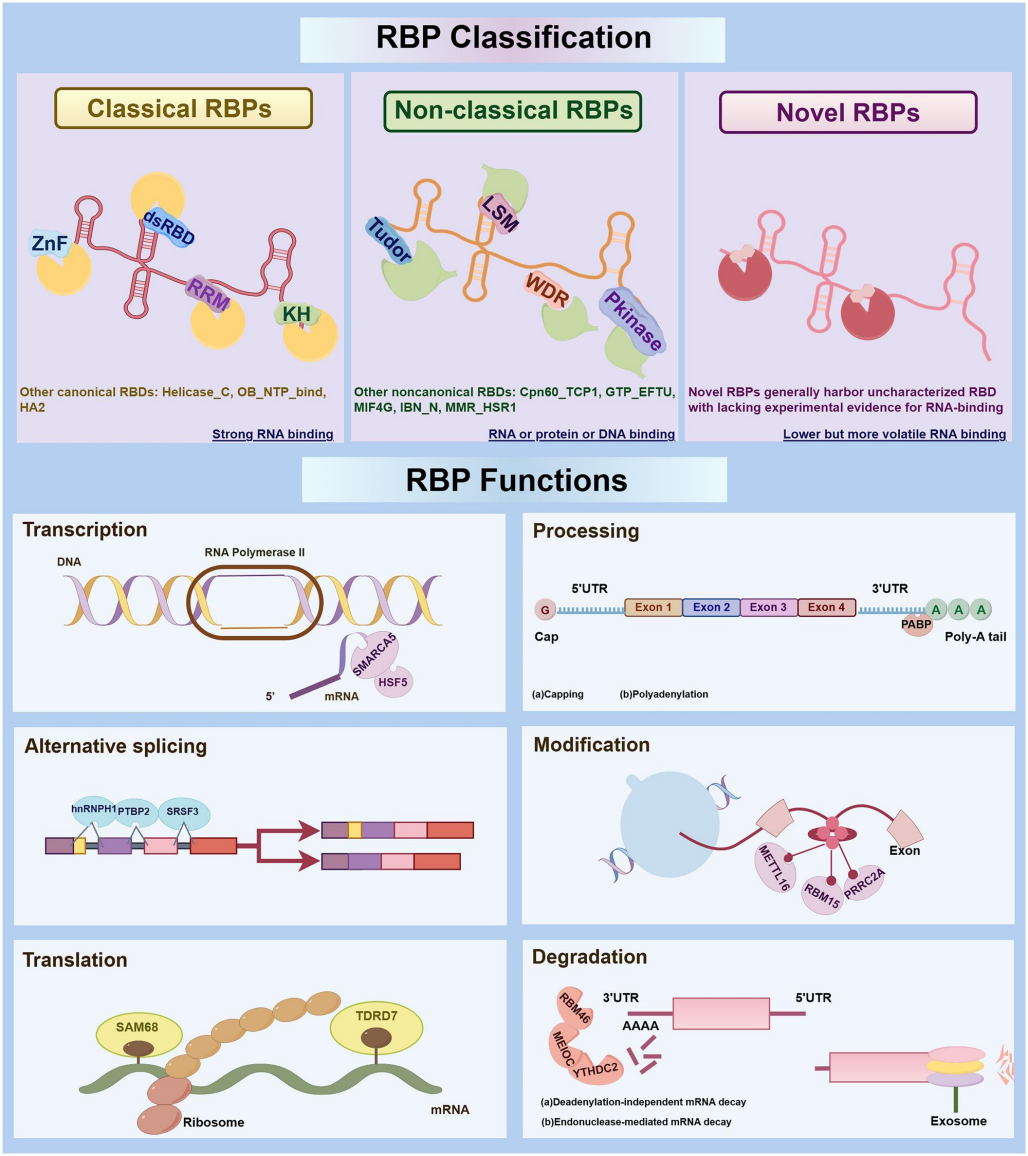
**Classification of RNA-binding proteins (RBPs) and their roles in RNA regulation.** **RBP classification**: classical, non-classical, and novel RBPs. **Classical RBPs** exhibit consistently strong RNA-binding activity and contain well-characterized RNA-binding domains (RBDs), such as the RNA recognition motif, double-stranded RNA-binding domain, K-homology, and zinc finger domains. **Non-classical RBPs** lack canonical RBDs but interact with RNA via noncanonical RBDs. These domains, such as WD40 repeat, Tudor, Like-Sm, and protein kinase domains, often possess the ability to bind not only RNA but also proteins or DNA. Compared to classical and non-classical RBP, **novel RBPs** generally harbor uncharacterized RBD with lacking experimental evidence for RNA-binding. **RBP Functions**: RBPs play essential roles in both transcriptional and post-transcriptional regulation of gene expression. At the transcriptional level, for example, HSF5 interacts with the chromatin remodeler SMARCA5, functioning as a meiosis-specific transcription factor essential for pachytene progression. Post-transcriptionally, RBPs regulate RNA metabolism, including processing, alternative splicing, modification, translation, and degradation. For instance, poly(A)-binding protein stabilizes transcripts via poly(A) tail binding; hnRNPH1 recruits PTBP2 and SRSF3 to modulate alternative splicing during germ cell development; METTL16, PRRC2A, and RBM15 mediate N6-methyladenosine deposition; DAZL and SAM68 regulate translational timing during spermatogenesis; and RBM46 also forms a conserved regulatory network with MEIOC and YTHDC2 to promote mitotic mRNA clearance, ensuring proper meiotic entry. Together, these multifaceted functions highlight the central roles of RBPs in maintaining RNA homeostasis and orchestrating gene expression during differentiation, development, and gametogenesis. dsRBD, double-stranded RNA-binding domain; KH, K-homology; LSM, Like-Sm; Pkinase, protein kinase; RRM, RNA recognition motif; WDR, WD40 repeat; ZnF, zinc finger.

RBPs interact with RNA molecules through specific RNA-binding domains (RBDs), enabling them to recognize and bind diverse RNA targets, including exons, introns, untranslated regions (UTRs), and non-coding RNAs such as microRNAs, small interfering RNAs (siRNAs), and small nucleolar RNAs. A total of 1542 RBP genes have been identified, accounting for ∼7.5% of all protein-coding genes in human (Gerstberger *et al.*, 2014; Agarwal *et al.*, 2021), highlighting their prevalence and functional significance. RBPs are categorized into three main categories based on their RNA-binding properties: classical RBPs, non-classical RBPs, and novel RBPs (Fig. 2). This classification reflects differences in domain architecture, evolutionary conservation, and modes of RNA engagement.

Classical RBPs typically possess well-characterized RBDs and exhibit consistently strong RNA-binding affinity, as demonstrated in multiple high-throughput interactome studies (Castello *et al.*, 2012, 2017; Hentze *et al.*, 2018; Li *et al.*, 2024c). Canonical RBDs include the RNA Recognition Motif (RRM), double-stranded RNA-binding domain (dsRBD), K-homology (KH), zinc finger (ZnF), and DEAD/DEAH-box helicase domains. Among these, the RRM is the most common, typically binding single-stranded RNA via its β-sheet surface. Representative classical RBPs like DAZL and BOULE utilize RRMs to regulate germ cell proliferation, meiosis, and spermatid maturation (Kee *et al.*, 2009). dsRBD binds to double-stranded RNA structures, regulating RNA silencing and editing. KH domain binds single-stranded RNA or DNA, contributing to mRNA stabilization and translational control. ZnF domain recognizes RNA secondary structures and mediates diverse functions, while DEAD/DEAH helicase domain facilitates RNA remodeling through ATP-dependent helicase activity.

Non-classical RBPs lack canonical RBDs, yet can interact with RNA via noncanonical RBDs. These include the WD40 repeat (WDR), Tudor, Like Sm (LSM), calponin homology (CH), and Protein kinase (Pkinase) domains (Li *et al.*, 2024c). Many of these domains were initially characterized for their roles in protein–protein interactions or chromatin regulation, but accumulating evidence indicates their involvement in RNA recognition under specific cellular contexts. Among these, WDR performs multiple functions through its ability to interact with distinct partners, both RNAs and proteins (Martinez-Salas and Francisco-Velilla, 2026). Tudor domain-containing proteins are implicated in piRNA biogenesis, chromatin remodeling, and DNA damage response (Pek *et al.*, 2012), while LSM proteins contribute to mRNA splicing, decapping, and RNA degradation (Okamoto *et al.*, 2016).

Compared to classical and non-classical RBP, novel RBPs generally harbor uncharacterized RBD with lacking experimental evidence for RNA-binding. They tend to display lower and more variable RNA-binding affinity (Li *et al.*, 2024c), which may reflect their dynamic access to stage-specific transcriptomes. These proteins have been identified through advanced high-throughput RNA–protein interactome profiling techniques, such as RNA Interactome Capture, Phenol Toluol Extraction, and Orthogonal Organic Phase Separation (Queiroz *et al.*, 2019; Urdaneta *et al.*, 2019; Li *et al.*, 2024c). The continuous discovery of novel RBPs is steadily expanding the known RNA interactome and opening new avenues for elucidating previously unrecognized regulators of gene expression, particularly those with roles in germ cell development and reproductive biology.

### Biological functions of RBPs in RNA metabolism

RBPs are key regulators of gene expression, orchestrating both transcriptional and post-transcriptional processes to ensure precise RNA metabolism. These versatile proteins bind to RNA molecules, regulating transcription, processing, alternative splicing, modification, translation, and degradation (Fig. 2). The multifaceted roles of RBPs underpin their critical contributions to cellular and developmental processes.

#### Transcriptional regulation by RBPs

Although RBPs are predominantly associated with post-transcriptional events, some also exert direct regulatory effects at the transcriptional level, influencing chromatin structure, transcription factor activity, and RNA polymerase recruitment (Chen and Huang, 2023).

Firstly, RBPs often interact with chromatin remodelers to regulate transcription. HSF5, a transcription factor with RNA-binding capacity, interacts with the chromatin remodeler SMARCA5 to facilitate transcriptional activation during meiotic progression. This highlights the contribution of RBPs to both chromatin remodeling and transcriptional activation (Liu *et al.*, 2024a; Luo *et al.*, 2024). Secondly, certain RBPs modulate transcription elongation by enhancing the processivity of RNA polymerase II, thereby supporting the efficient expression of genes, particularly those with long and intron-rich transcripts. TAF15, an RBP associated with the TFIID complex, has been shown to stabilize the transcriptional machinery and promote transcription elongation (DeJong *et al.*, 2021). Notably, many TAF15-regulated genes are highly expressed in reproductive tissues, suggesting its critical role in sustaining the transcriptional output necessary for germ cell development and function. Thirdly, RBPs can act as transcriptional co-regulators. For example, hnRNPU has been reported to bind enhancer regions and recruit transcriptional activators, thereby bridging transcription initiation with subsequent RNA processing events. This emphasizes the integrative roles of RBPs in coupling transcription with downstream RNA regulatory processes (Wen *et al.*, 2021).

#### Post-transcriptional regulation by RBPs

RBPs play a dominant role in post-transcriptional regulation, influencing the fate and function of RNA molecules through various mechanisms.

*RNA processing*. RBPs are central to RNA maturation events, including 5′ capping, cleavage, and polyadenylation. Poly(A)-binding protein (PABP) binds the 3′-UTR of polyadenylated transcripts to enhance mRNA stability and translation efficiency (Biziaev *et al.*, 2024). Similarly, CPSF4, an RBP involved in the cleavage and polyadenylation complex, ensures proper transcript maturation for subsequent translation (Lee *et al.*, 2021).

*Alternative splicing*. Testis-specific alternative splicing is a hallmark of spermatogenesis, with RBPs serving as key regulators (Tao *et al.*, 2024; Li *et al.*, 2025). hnRNPH1 facilitates exon inclusion by recruiting splicing factors such as PTBP2 and SRSF3, generating isoforms essential for developmental progression (Feng *et al.*, 2022b). Aberrant alternative splicing can result in dysfunctional isoforms, contributing to infertility and other disease phenotypes.

*RNA modification*. RBPs regulate chemical modifications of RNA, such as N6-methyladenosine (m6A) modification, which impacts RNA stability, splicing, and translation. METTL16, RBM15, and PRRC2A function as m6A ‘writers’ by catalyzing methyl group addition on specific mRNAs, thereby regulating transcript stability and functionality (Tan *et al.*, 2023b; Ma *et al.*, 2024; Park *et al.*, 2024). m6A ‘readers’ like YTHDF protein family interpret these modifications to mediate RNA degradation or promote translation (Zou and He, 2024).

*RNA localization and translation regulation*. RBPs precisely control when and where RNA is translated to ensure proper developmental progression. SAM68 has been shown to regulate stage-specific translation of target mRNAs by recruiting polysome during spermatogenesis (Paronetto *et al.*, 2009). In addition, TDRD7 directs mRNAs to distinct subcellular compartments where localized translation supports specific developmental processes (Tanaka *et al.*, 2011).

*RNA stability and degradation*. RBPs maintain transcriptome integrity by stabilizing essential RNAs and targeting defective or unnecessary transcripts for degradation. ELAVL1 stabilizes its target mRNAs by binding to their 3′-UTRs. Notably, many of these targets encode transcriptional regulators (Rothamel *et al.*, 2021). RBM46, functions as part of the YTHDC2/MEIOC complex, a key regulator of meiotic entry. RBM46 binds to mitotic transcripts and facilitates their degradation, thereby contributing to the transition from mitosis to meiosis in germ cells (Qian *et al.*, 2022).

Taken together, RBPs are indispensable for both transcriptional and post-transcriptional regulation, acting as master regulators of RNA metabolism. Their ability to modulate transcription, RNA processing, stability, and localization reflects their central role in maintaining gene expression homeostasis. Understanding these mechanisms not only illuminates the molecular basis of cellular regulation but also provides critical insights into RBP-associated pathologies.

## RBPs-related genes in human male infertility

With the rapid advancement of next-generation sequencing technologies and progress in reproductive genetics, numerous pathogenic variants in RBP genes have been implicated in male infertility disorders, including azoospermia, oligozoospermia, teratozoospermia, and asthenozoospermia (Table 1). These discoveries underscore the critical roles of RBPs in orchestrating spermatogenesis. In parallel, many VUS in RBP genes have been identified in affected individuals, posing challenges for clinical interpretation while providing opportunities to advance our understanding of their functional significance (Table 2).

**Table 1.** Identification of pathogenic variants in RNA-binding protein genes in human male infertility.

Mutant genes	Variants	Changes in amino acids	Zygosity	Domains	ACMG classification	Studied population	Mutant phenotypes	Ref.
**Azoospermia and oligozoospermia**								
*AKAP4*	c.1286G>A	p.Arg429His	Hemi	Downstream of the PKA-RI-alpha subunit binding domain	P	Two siblings suffering from NOA	NOA	Wei *et al.* (2023)
*AR*	c.1106T>C	p.Leu369Pro	Hemi	Upstream of the ZnF domain	LP/P	521 individuals presenting idiopathic SPGF and 323 normozoospermic men	NOA	Lillepea *et al.* (2024)
	c.1286C>A	p.Ala429Asp	Hemi	Upstream of the ZnF domain				
	c.1723C>G	p.Leu575Val	Hemi	ZnF domain				
*CCER1*	c.157C>T	p.Arg53*	Het	IDRs	P	Five NOA patients among 620 NOA patients	NOA	Qin *et al.* (2023)
	c.358_388del	p.Cys120fs	Het	Outside the CCs				
	c.534G>A	p.Trp178*	Het	Outside the CCs				
*DAZL*	c.197T>A	p.Val66Glu	Het	RRM domain	LP/P	200 infertile male	Oligo-/azoospermia	Li *et al.* (2024c)
	c.218_220insG	p.Asp74fs	Het	RRM domain				
*DDX3Y*	c.428dup	p.Glu145fs	Hemi	Upstream of the DEAD/DEAH-box helicase domain	LP	Three unrelated infertile men among 1655 well-characterized men	NOA, SCOS	Dicke *et al.* (2023)
	c.1230_1231del	p.Asn412fs	Hemi	C-terminal helicase domain				
	c.1272dup	p.Lys425Ter	Hemi	C-terminal helicase domain				
	c.1609 + 1del	p.Gly537Alafs*12	Hemi	C-terminal helicase domain				
*DDX25* *(GRTH)*	c.1129C>T	p.Arg377Ter	Hom	DEAD/DEAH-box helicase domain	P	One man among 96 unrelated men originating from North Africa	NOA	Kherraf *et al.* (2022)
*DHX37*	c.923G>A	p.Arg308Gln	Het	DEAD/DEAH-box helicase domain	P	Four 46, XY patients with partial gonadal dysgenesis	Oligo-/azoospermia	de Oliveira *et al.* (2023)
*DMRT1*	c.395G>A	p.Gly132Asp	Het	Downstream of the DM DNA-binding domain	LP	521 individuals presenting idiopathic primary SPGF	Oligozoospermia, cryptozoospermia	Lillepea *et al.* (2024)
	c.425C>T	p.Ala142Val	Het	Downstream of the DM DNA-binding domain				
	c.344T>A	p.Met115Lys	Hemi	DM DNA-binding domain	LP	647 crypto-/azoospermic men	NOA	Wyrwoll *et al.* (2023)
*FKBP6*	c.508_529dup	p.Phe177fs	Hom	TPR 1	P	2699 men with SPGF	NOA	Wyrwoll *et al.* (2022a)
	c.589-2A>G	p.Ala197Glyfs*31	Hom	TPR1				
	c.610C>T	p.Arg204Ter	Hom	TPR1				
	c.832C>T	p.Arg278Ter	Het	TPR3				
*GPAT2*	c.1879C>T c.1954C>T	p.Arg627Trp p.Arg652Ter	Hom Het	Glycerol-3-phosphate acyltransferase C-terminal region Glycerol-3-phosphate acyltransferase C-terminal region	LP/P	39 infertile men	NOA, SCOS	Stallmeyer *et al.* (2024)
*GTSF1*	c.221_222del	p.Arg74Lysfs*4	Hom	ZnF domain 1	LP/P	39 infertile men	NOA, spermatogonial arrest	
	c.97C>A	p.His33Asn	Hom	ZnF domain 2				
*HFM1*	c.3490C>T	p.Gln1164Ter	Hom	Downstream of the SEC63 domain	P	Two patients among 51 patients with NOA	NOA, meiotic arrest	Tang *et al.* (2021)
	c.1355G>A	p.Arg452Gln	Hom	DEAD/DEAH-box helicase domain	P	Three Chinese patients with NOA and two brothers with NOA from the Pakistani family		Xie *et al.* (2022)
	c.1472T>C	p.Leu491Pro	Hom	Downstream of the ATP-binding domain				
	c.2487_2491del	p.Lys829fs	Het	SEC63 domain				
	c.2562_2563del	p.Glu856fs	Het	SEC63 domain				
	c.4126del	p.Glu1376fs	Het	Downstream of the SEC63 domain				
*HIPK4*	c.1099C>T	p.Arg367Cys	Het	Downstream of the Pkinase domain	LP	10 patients among 620 NOA patients	NOA	Liu *et al.* (2022b)
	c.1468A>T	p. Lys490*	Het	IDRs				
*KCTD19*	c.1897C>T	p.Gln633*	Hom	Downstream of the second BTB/POZ domain	P	Two unrelated infertile Chinese men and a consanguineous Pakistani family with three infertile brothers	NOA, meiotic arrest	Liu *et al.* (2023b)
	c.2005C>T	p.Gln669*	Hom	IDRs				
	c.G628A	p.Glu210Lys	Hom	Downstream of the first BTB/POZdomain	LP	Five infertile males from three unrelated families among 536 individuals with idiopathic oligozoospermia	OAT	Wang *et al.* (2023a)
	c.C893T	p.Pro298Leu	Hom	Downstream of the first BTB/POZdomain				
	c.G2309A	p.Gly770Asp	Hom	Downstream of the second BTB/POZdomain				
*MAEL*	c.799C>T	p.Arg267Ter	Het	Maelstrom domain	LP/P	39 infertile men	NOA, spermatogonial arrest	Stallmeyer *et al.* (2024)
	c.908 + 1G>C	p.Cys283_Ala303del	Het	Maelstrom domain				
*MAGEB4*	c.1041A>T	p.Ter347Cys	Hom	Downstream of the melanoma-associated antigen domain	LP	Two azoospermic brothers among a consanguineous Turkish family comprising nine siblings	NOA	Okutman *et al.* (2017)
*MOV10L1*	c.2447G>T	p.Ser816Ile	Het	AAA domain	LP/P	456 patients with male infertility	NOA	Li *et al.* (2022a)
	c.3094_3097del	p.Pro1032fs	Het	AAA domain				
	c.2179 + 3A>G	p.Asn691*	Hom	Downstream of the Helicase MOV-10, beta-barrel domain	LP	39 infertile men	NOA, spermatogonial arrest	Stallmeyer *et al.* (2024)
	c.2485G>A	p.Ala829Thr	Hom	AAA domain	LP	26 patients with idiopathic NOA	NOA	Ghieh *et al.* (2022a)
*NANOS2*	c.208C>T	p.Gly70Arg	Het	NANOS RNA-binding domain	P	8 consanguineous families	NOA	Fakhro *et al.* (2018)
*PDHA2*	c.679A>G	p.Met227Val	Hom	TED consensus domain	P	96 NOA-affected individuals	NOA	Kherraf *et al.* (2022)
*PIWIL1*	c.688C>T	p.Arg230Ter	Hom	Linker domain 1	LP	39 infertile men	NOA, sound spermatid arrest	Stallmeyer *et al.* (2024)
*PIWIL2*	c.731_732delAT	p.His244ArgfsTer31	Hom	PAZ domain	P	A patient from a consanguineous family	NOA, SCOS	Wang *et al.* (2022c)
	c.727_729del	p.Tyr243del	Hom	PAZ domain	LP	2 infertile men among 285 male patients	NOA	Alhathal *et al.* (2020)
*PRM3*	c.178G>T	p.Gly60Trp	Het	IDRs	LP	125 infertile men with either idiopathic NOA or oligozoospermia	NOA or oligozoospermia	Imken *et al.* (2009)
*RBMXL2*	c.301dup	p.Ser101LysfsTer29	Hom	IDRs	P	A man with an NOA	NOA, meiotic arrest	Ghieh *et al.* (2022b)
*SPINK2*	c.56-3C>G	Create a new splice acceptor site	Hom	Outside the Kazal-like domain	P	Two azoospermic brothers	Azoospermia	Kherraf *et al.* (2017)
*SYCE1*	c.689_690del	p.Phe230fs	Hom	CCs	P	An NOA patient	NOA	Feng *et al.* (2022a)
	c.1_1113del	p.1del371aa	Hom	Outside the CCs		One man among 235 idiopathic NOA patients		Krausz *et al.* (2020)
*SYCP2*	c.2022_2025del	p.Lys674fs	Het	IDRs	LP/P	Male Infertility Cohort Participants	NOA	Schilit *et al.* (2020)
	c.2793_2797del	p.Lys932fs	Het	Downstream of the Synaptonemal complex 2 Spt16M-like domain				
	c.3067_3071del	p.Lys1023fs	Het	Downstream of the Synaptonemal complex 2 Spt16M-like domain				
*TDRD1*	c.887C>A	p.Ser296Tyr	Hom	Outside the Tudor domain	LP	39 infertile men	Azoospermia, meiotic arrest	Stallmeyer *et al.* (2024)
*TDRD6*	c.1825G>T	p.Gly609X	Hom	Outside the Tudor domain	P	A 38-year-old patient from a consanguineous family	OAT	Sha *et al.* (2018b)
*TDRD7*	c.324_325insA	p.Thr110Asnfs*30	Hom	Outside the Tudor domain	P	Two brothers among 176 patients with NOA or CC	NOA	Tan *et al.* (2019)
	c.688_689insA	p.Tyr230X	Hom	Outside the Tudor domain				
*TDRD9*	c.3148dup	p.Val1050fs	Hom	Outside the Tudor domain	LP	39 infertile men	Extreme oligozoospermia	Stallmeyer *et al.* (2024)
	c.3716 + 3A>G	p.Ser1208Leufs*5	Hom	Outside the Tudor domain				
	c.958delC	p.His320Ilefs*28	Het	Downstream of the DEAD/DEAH-box helicase domain	P	One proband from a Chinese family	Oligozoospermia	Wang *et al.* (2024b)
*TEX11*	c.313C>T	p.Arg105*	Hom	Upstream of the meiosis protein SPO22/ZIP4 like motif	P	Three NOA patients from three families	NOA	Song *et al.* (2023b)
	c.427A>C	p.Lys143Gln	Hom	Upstream of the SPO22 motif				
	c.2575G>A	p.Gly859Arg	Hom	Downstream of the TPR-containing regions				
	c.511A>G	p.Met171Val	Het	TPR1 domain	P	49 men of European descent who had azoospermia	Azoospermia, meiotic arrest	Yatsenko *et al.* (2015)
	c.652del237bp	p.218del79aa	Hemi	Meiosis-specific SPO22 motif				
	c.450C>T	p.Ala150Ala spl d	Het	TPR1 domain	P	7 of 289 men with azoospermia		
	c.792 + 1G>A	p.Leu264 spl d	Hemi	Meiosis-specific SPO22 motif				
	c.2092G>A	p.Ala698Thr	Het	TPR3 domain				
*TEX14*	c.1003C>T	p.Arg335*	Hom	Pkinase domain	LP/P	521 individuals presenting idiopathic primary SPGF	NOA, SCOS	Lillepea *et al.* (2024)
	c.1021C>T	p.Arg341Ter	Hemi	Pkinase domain	P	647 crypto-/azoospermic men	NOA	Wyrwoll *et al.* (2023)
	c.1508T>C	p.Leu503Pro	Hemi	Pkinase domain				
	c.2962C>T	p.Gln988Ter	Hom	IDRs				
	c.3186C>A	p.Tyr1062Ter	Hemi	Downstream of the Pkinase domain				
	c.3620_3624del	p.Pro1207fs	Hom	Downstream of the Pkinase domain				
	c.3687C>A	p.Ser1255fs	Hemi	Downstream of the Pkinase domain				
	c.3763_3766del	p.Cys1229Ter	Hemi	Downstream of the Pkinase domain				
	c.2668_2678del	p.Ser890fs	Hom	Downstream of the Pkinase domain	P	Siblings from three families who reported familial infertility	NOA	Gershoni *et al.* (2017)
	c.254G>T	p.Arg85Leu	Hom	Upstream of the ankyrin repeat 3	P	One family among eight consanguineous families	NOA	Fakhro *et al.* (2018)
*TSPYL5*	c.1057A>G	p.Thr353Ala	Het	TED consensus domain	LP	1558 Han Chinese men with different spermatogenic conditions	Azoospermia	Yang *et al.* (2018)
*USP9Y*	c.6537T>A	p.Tyr2179Ter	Hemi	Downstream of the ubiquitin specific protease domain	P	1 male among 69 Jordanian men with NOA	NOA	Batiha *et al.* (2022)
*WT1*	c.1178T>A	p.Met393Lys	N.R.	Classic ZnF domain	LP	521 individuals presenting idiopathic primary SPGF	NOA or oligozoospermia	Lillepea *et al.* (2024 **)**
	c.1193T>A	p.Met398Lys	N.R.	Classic ZnF domain				
	c.1238T>A	p.Met413Lys	N.R.	Classic ZnF domain				
	c.1244T>A	p.Met415Lys	Het	Classic ZnF domain				
*YTHDC2*	c.962G>C	p.Arg321Thr	Hom	DEAD/DEAH-box helicase domain	LP	Two NOA affected siblings from a consanguineous Pakistani family	NOA	Tian *et al.* (2024)
*ZFP318 (ZNF318)*	c.493G>A	p.Arg165Ter	Hom	IDRs	LP	47 idiopathic infertile men	NOA, OAT	Sudhakar *et al.* (2023)
*ZMYND15*	c.1520_1523del	p.Lys507fs	Hom	MSS51 C-terminal domain	P	Three azoospermic brothers	NOA	Cannarella *et al.* (2025)
	c.1209T>A	p.Tyr403Ter	Hom	Downstream of the MYND-type ZnF domain	P	219 unrelated Chinese patients	Severe oligozoospermia	Hu *et al.* (2021)
	c.1622_1636delinsCCAC	p.Leu541fs	Hom	MSS51 C-terminal domain				
	c.1650del	p.Glu551fs	Hom	MSS51 C-terminal domain				
**Teratozoospermia: Acrosome defects, ASS, and Globozoospermia**								
*ACTL7A*	c.733G>A	p.Ala245Thr	Hom	Actin-like ATPase domain	P	Two infertile brothers	Acrosome defects	Xin *et al.* (2020)
	c.463C>T	p.Arg155Ter	Het	Actin-like ATPase domain	P	A non-consanguineous family whose son was affected by total fertilization failure		Wang *et al.* (2021)
	c.1084G>A	p.Gly362Arg	Het	Actin-like ATPase domain				
*ACR*	c.167G>A	p.Trp56_Ser421del	Hom	Peptidase S1 domain	LP	Two primary infertile brothers whose parents were first cousins		Hua *et al.* (2023)
*CCIN*	c.1294C>T	p.Arg432Trp	Het	Kelch 4 repeat	P	1 patient among 126 patients with teratozoospermia	Severe sperm head malformation	Fan *et al.* (2022)
	c.1341C>A	p.Cys447Ter	Het	Kelch 4 repeat				
	c.125A>T	p.His42Leu	Hom	BTB domain		Two infertile brothers from a consanguineous family		
	c.853G>A	p.Gly285Ser	Hom	Kelch 1 repeat	P	One patient among 15 cases of unexplained globozoospermia	Globozoospermia, acrosomal hypoplasia	Oud *et al.* (2020)
*DPY19L2*	c.153_189del	p.Trp52SerfsTer7	Hom	IDRs	P	69 globozoospermic patients	Globozoospermia	Celse *et al.* (2021)
	c.869G>A	p.Arg290His	Het	TED consensus domain				
	c.892C>T	p.Arg298Cys	Het	TED consensus domain				
	c.1580 + 1G>A	p.512_527delfsTer5	Hom	TED consensus domain				
	c.1840G>T	p.Glu614Ter	Hom	Outside the TED consensus domain				
*GGN*	c.416_437del	p.Leu139fs	Hom	IDRs	P	1 patient among 23 globozoospermic patients	Globozoospermia	
	c.1271del	p.Gly424fs	Hom	IDRs	P	2 patients among 15 cases of unexplained globozoospermia	Globozoospermia	Oud *et al.* (2020)
*SPACA1*	c.53G>A	p.Trp18Ter	Hom	Outside the TED consensus domain	P	Two globozoospermic brothers and their consanguineous parents	Globozoospermia	Chen *et al.* (2021b)
*SPATA20*	c.1957 + 2T>A	Abrogate the consensus splice donor site	Hom	Six-hairpin glycosidase domain	P	One non-consanguineous infertile patient originating from Southeast Europe	Globozoospermia	Martinez *et al.* (2023)
*TSGA10*	c.211del	p.A71Hfs*12	Hom	TED consensus domain	P	A 27 years old infertile male from a consanguineous family	ASS	Sha *et al.* (2018a)
*ZPBP*	c.931C>T	p.Gln311*	Hom	Zona-pellucida-binding protein C-terminal EGF-like domain	P	15 cases of infertile patients	Globozoospermia	Oud *et al.* (2020)
**Asthenozoospermia: MMAF and PCD**								
*AKAP3*	c.44G>A	p.Cys15Tyr	Hom	Upstream of the PKA-RII subunit binding domain	LP/P	150 Han Chinese men with asthenoteratozoospermia	MMAF	Liu *et al.* (2023a)
	c.2286_2287del	p.His762Glnfs*22	Hom	Downstream of the PKA-RII subunit binding domain				
*CCDC39*	c.1007_1010del	p.Lys336Argfs*19	Het	CCs	P	An infertile male patient with PCD	MMAF	Aprea *et al.* (2023)
	c.1072del	p.Thr358Glnfs*3	Het	CCs				
	c.983 T > C	p. Leu328Pro	Hom	CCs	P	Two PCD-affected siblings of a consanguineous family	MMAF	Chen *et al.* (2021a)
*CCDC40*	c.901C>T	p.Arg301*	Het	CCs	LP/P	Infertile patients with PCD	MMAF	Liu *et al.* (2021)
	c.2065_2068dup	p.Ala690Glyfs*67	Het	CCs				
	c.248del	p.Ala83Valfs*84	Het	IDRs	LP	Two infertile male patients with PCD		Aprea *et al.* (2023)
	c.736_755dup	p.Ser252Argfs*43	Het	IDRs				
	c.1675G>T	p.Glu559*	Hom	CCs				
*DNAH1*	c.2602C>T	p.Arg868X	Het	Upstream of the N-terminal region	P/LP	Forty-one Chinese patients with MMAF	MMAF	Yu *et al.* (2021)
	c.4552C>T	p.Gln1518X	Het	Downstream of the N-terminal region				
	c.9850G>A	p.Glu3284Lys	Het	AAA5 region				
	c.11726_11727del	p.Pro3909fs*33	Hom	Downstream of the AAA 6 region				
	c.12287G>T	p.Arg4096Leu	Het	Downstream of the AAA 6 region				
	c.3860T>G	p.Val1287Gly	Hom	N-terminal region	P	5 patients among two families		Amiri-Yekta *et al.* (2016)
*DNAH2*	c.5770C>T	p.Arg1924Cys	Het	AAA 1 region	P	4 patients among 38 patients with primary infertility and diagnosed with MMAF	MMAF	Li *et al.* (2019b)
	c.6960C>A	p.Ser2320Arg	Het	Downstream of the AAA 2 region				
	c.9298C>T	p.Arg3100Trp	Hom	TPR 3 repeat				
	c.11500C>T	p.Arg3834Ter	Het	AAA 6 region				
	c.11503T>C	p.Ser3835Pro	Het	AAA 6 region				
*DNAH3*	c.5143G>A	p.Gly1715Ser	Het	AAA 2 region	LP/P	3 patients among 432 infertile Chinese men	Dislocated mitochondrial sheath	Meng *et al.* (2024)
	c.6973T>C	p.Phe2325Leu	Het	Downstream of the AAA 3 region				
	c.10260 G > A	p.Trp3420X	Het	Downstream of the AAA 5 region				
	c.10439G>A	p.Arg3480Gln	Het	Downstream of the AAA 5 region				
*DNAH8*	c.6158_6159insT	p.Gly2054Trpfs*50	Hom	Downstream of the AAA 1 region	LP	Two infertile brothers from a consanguineous Pakistani family	MMAF	Dil *et al.* (2023)
	c.6689A>G	p.Lys2230Arg	Het	AAA 2 region	P	2 patients among 90 Chinese men		Liu *et al.* (2020)
	c.9427C>T	p.Arg3143Cys	Het	CCs				
	c.11771C>T	p.Thr3924Met	Het	AAA 6 region				
	c.6962_6968del	p.His2321Profs*4	Hom	Downstream of the AAA 2 region		1 patient among 167 men from France, Iran, and North Africa		
	c.12721G>A	p.Ala4241Thr	Het	Downstream of the AAA 6 region				
*DNAH17*	c.1293_1294del	p.Tyr431*	Het	N-terminal region	LP	5 infertile men	MMAF	Whitfield *et al.* (2019)
	c.5486G>A	p.Cys1829Tyr	Hom	AAA 1 region				
	c.7994_8012del	p.Gly2665fs	Het	AAA 3 region				
	c.10486_10497dup	p.Val3496_Pro3499dup	Het	AAA 5 region				
	c.10784T>C	p.Leu3595Pro	Het	AAA 5 region				
*FSIP2*	c.5480A>T	p.Asp1827Val	Het	Outside the CCs and IDRs	LP	Four asthenoteratozoospermic patients	Flagellar defects, and acrosomal hypoplasia	Zheng *et al.* (2023)
	c.9056T>C	p.Ile3019Thr	Het	Outside the CCs and IDRs				
	c.10823T>C	p.Leu3608Ser	Het	Outside the CCs and IDRs				
	c.17798C>T	p.Ser5933Phe	Het	Outside the CCs and IDRs				
	c.910del	p.Gln304fs	Hom	Outside the CCs and IDRs	P	Four unrelated individuals from 78 infertile men	MMAF	Martinez *et al.* (2018)
	c.1606_1607insTGT	p.Lys536fs	Hom	Outside the CCs and IDRs				
	c.2282dup	p.Asn761fs	Hom	Outside the CCs and IDRs				
	c.16389_16392delAATA	p.Asn5465fs	Hom	Outside the CCs and IDRs				
	c.4574C>A	p.Ser1525*	Het	Outside the CCs and IDRs	LP/P	Three unrelated individuals from a cohort of 105 patients with asthenoteratozoospermia		Lv *et al.* (2023)
	c.8104dup	p.Leu2702Profs*44	Het	Outside the CCs and IDRs				
	c.9043G>A	p.Glu3015Lys	Het	Outside the CCs and IDRs				
	c.9234dup	p.Leu3079Thrfs*6	Het	Outside the CCs and IDRs				
	c.10247dup	p.Glu3417Glyfs*12	Het	Outside the CCs and IDRs				
	c.1907C>A	p.Ser636*	Hom	Outside the CCs and IDRs	P	40 unrelated Han Chinese men affected with MMAF		Liu *et al.* (2019b)
	c.8030_8031insA	p.Thr2680Asnfs*9	Hom	Outside the CCs and IDRs				
*MDC1*	c.472C>T	p.Gln158*	Het	Upstream of the forkhead-associated domain	LP	One patient from Australian cohort with severe sperm motility disorders	Severe sperm motility disorders	Oud *et al.* (2021)
	c.2134C>T	p.Gln712*	Het	Downstream of the forkhead-associated domain				
*RSPH1*	c.680dup	p.Pro228Alafs*15	Hom	IDRs	P	Two infertile male patients with PCD	MMAF	Aprea *et al.* (2023)
*STK36*	c.1399delG	p.Glu467Argfs*13	Hom	Armadillo repeat	P	One PCD-affected individual	PCD, reduced sperm motility	Edelbusch *et al.* (2017)
*TTC21A*	c.3116 + 5G>T	intron variant	Hom	TPR 16	P	Two Tunisian cases from an independent MMAF cohort	Asthenoteratozoospermia, MMAF	Liu *et al.* (2019a)
	c.341A>G	p.Tyr114Cys	Het	TPR 2		3 males among 65 Han Chinese men with MMAF		
	c.716 + 1G>A	p.Ile240*	Hom	TPR 5				
	c.2329C>T	p.Gln777Ter	Het	TPR 10				
	c.2563del	p.Ala854_Val855insTer	Hom	TPR 12				
*WDR19*	c.3811A>G	p.Lys1271Glu	Hom	First ZnF domain	P	One infertile male among 65 asthenoteratospermia individuals	Asthenoteratospermia, MMAF	Ni *et al.* (2020)
*ZMYND10* *(DNAAF7)*	c.47T>G	p.Val16Gly	Hom	Upstream of the MYND-type ZnF domain	LP	One male from study cohort with PCD symptoms	MMAF	Aprea *et al.* (2021)

Gene variants were obtained from the ClinVar and PubMed databases, while domain data were sourced from UniProt and InterPro databases. RRM, RNA recognition motif; AAA, ATPases Associated with diverse cellular Activities; ACMG, American College of Medical Genetics and Genomics; ASS, acephalic spermatozoa syndrome; CCs, Coiled coils; BTB, Bric-à-brac, tramtrack, broad complex; Hom, Homozygous; Het, Heterozygous; Hemi, Hemizygous; IDRs, intrinsically disordered regions; LP, likely pathogenic; MMAF, morphological abnormalities of the flagella; NOA, non-obstructive azoospermia; N.R., no reported. OAT, oligo-astheno-teratozoospermia; P, Pathogenic; Pkinase, protein kinase; PCD, primary ciliary dyskinesia; PAZ, Piwi/Argonaute/Zwille; POZ, Poxvirus and zinc finger; SCOS, Sertoli cell–only syndrome; SPGF, spermatogenic failure; TED, TRAF-EDD; TPR, tetratricopeptide repeat; ZnF, zinc finger.

**Table 2.** Identification of variants of uncertain significance in RNA-binding protein genes in human male infertility.

Mutant genes	Variants	Changes in amino acids	Zygosity	Domains	ACMG classification	Studied population	Mutant phenotypes	Ref.
**Azoospermia and oligozoospermia**								
*DDX4*	c.1532C>T	p.Ala511Val	Hom	Downstream of the DEAD/DEAH-box helicase domain	VUS	39 infertile men	Round spermatid arrest	Stallmeyer *et al.* (2024)
*DMRT1*	c.15G>C	p.Glu5Asp	Het	IDRs	VUS	Eleven infertile men with severely impaired spermatogenesis	Cryptozoospermia or azoospermia	Emich *et al.* (2023)
	c.59C>A	p.Pro20His	Het	IDRs				
	c.240G>C	p.Arg80Ser	Het	DM DNA-binding domain				
	c.308A>G	p.Lys103Arg	Het	DM DNA-binding domain				
	c.436C>G	p.Leu146Val	Het	Downstream of the DM DNA-binding domain				
	c.1054C>A	p.Leu352Ile	Het	IDRs				
	c.1108G>A	p.Glu370Lys	Het	IDRs				
	c.671A>G	p.Asn224Se	Het	Downstream of the DM DNA-binding domain	VUS	171 patients with cryptozoospermia or NOA	NOA, SCOS	Araujo *et al.* (2020)
*DAZL*	c.185G>C	p.Arg62Thr	N.R.	RRM domain	VUS	200 infertile men	Oligo-/azoospermia	Li *et al.* (2024c)
*DHX37*	c.1399C>G	p.Leu467Val	Het	DEAD/DEAH-box helicase domain	VUS	Four 46, XY patients with partial gonadal dysgenesis	Oligo-/azoospermia	de Oliveira *et al.* (2023)
	c.2995G>A	p.Val999Met	Het	Downstream of the DEAD/DEAH-box helicase domain				
*GPAT2*	c.1130A>G	p.His377Arg	Het	Glycerol-3-phosphate acyltransferase C-terminal region	VUS	39 infertile men	Azoospermia, SCOS	Stallmeyer *et al.* (2024)
	c.1388C>T	p.Thr463Met	Hom	Glycerol-3-phosphate acyltransferase C-terminal region				
	c.146G>A	p.Arg49His	Hom	Outside the TED consensus domain	VUS	39 infertile men	Extreme oligozoospermia	
*HFM1*	c.2681-3T>A	p.Trp894Cysfs*44	Hom	SEC63 domain	VUS	Two siblings from a Chinese family	NOA	Yao *et al.* (2023)
	c.3470G>A	p.Cys1157Tyr	Hom	Downstream of the SEC63 domain	VUS	Two patients among 51 patients with NOA	NOA, Meiotic arrest	Tang *et al.* (2021)
*HSF5*	c.586C>T	p.Arg196Cys	Hom	Downstream of the ‘Winged helix’ DNA-binding domain	VUS	Two infertile siblings from a consanguineous family	Cryptozoospermia, meiotic arrest	Liu *et al.* (2024a)
*HIPK4*	c.49A>C	p.Leu17Val	Het	Pkinase domain	VUS	10 patients among 620 NOA patients	NOA	Liu *et al.* (2022b)
	c.376G>A	p.Arg126Met	Het	Pkinase domain				
	c.817A>G	p.Thr273Ala	Het	Pkinase domain				
	c.1295T>C	p.Leu432Pro	Het	Downstream of the Pkinase domain				
	c.1772T>C	p.Leu591Pro	Het	IDRs				
*KLHL10*	c.242A>T	p.Asn81Ile	Het	BTB domain Kelch 3 repeat	VUS	Two patients among 16 patients with NOA from Ribeirao Preto, Brazil	NOA, SCOS	Araujo *et al.* (2020)
	c.887T>C	p.Ile396Thr	Het					
*MOV10L1*	c.2258T>C	p.Val753Ala	Hom	Downstream of the helicase MOV-10, beta-barrel domain	VUS	39 infertile men	Azoospermia	Stallmeyer *et al.* (2024)
	c.3115G>A	p.Glu1039Lys	Hom	Downstream of the helicase MOV-10, beta-barrel domain				
	c.3268G>T	p.Val1090Phe	Hom	Downstream of the helicase MOV-10, beta-barrel domain				
	c.2542G>A	p.Gly848Arg	Hom	Downstream of the helicase MOV-10, beta-barrel domain	VUS	456 patients with male infertility	NOA	Li *et al.* (2022a)
*PIWIL2*	c.839A>C	p.Tyr280Ser	Hom	Upstream of the PAZ domain	VUS	39 infertile men	Azoospermia, SCOS	Stallmeyer *et al.* (2024)
	c.1697G>A	p.Arg566His	Hom	Downstream of the PAZ domain				
	c.727_729del	p.Tyr243del	Hom	Upstream of the PAZ domain	VUS	2 infertile men among 285 male patients	NOA	Alhathal *et al.* (2020)
*PRM3*	c.65C>T	p.Thr22Met	Het	IDRs	VUS	125 infertile men with either idiopathic azoospermia or oligozoospermia	Azoospermia or oligozoospermia	Imken *et al.* (2009)
	c.258C>G	p.His86Gln	Het	IDRs				
	c.299G>A	p.Arg100Gl	Het	IDRs				
*RBM5*	c.217G>A	p.Glu73Lys	Het	IDRs	VUS	521 individuals presenting idiopathic primary SPGF	NOA, cryptorchidism, or oligozoospermia	Lillepea *et al.* (2024)
*RPL10L*	c. 257A>C	p.His86Pro	Hom	TED consensus domain	VUS	Two brothers with the severe oligozoospermia phenotype	Severe oligozoospermia	Tu *et al.* (2020)
*RNF126*	c.202G>A	p.Vla68Met	Het	Downstream of the C4-type ZnF domain	VUS	193 patients diagnosed with oligoasthenoteratozoospermia or NOA	NOA or OAT	Liu *et al.* (2024b)
	c.722G>A	p.Arg241His	Het	RING-type ZnF domain				
	c.757G>A	p.Asp253Asn	Het	RING-type ZnF domain				
	c.782A>C	p.Glu261Ala	Het	RING-type ZnF domain				
*SYCE1*	c.373A>G	p.Arg125Gly	Hom	CCs	VUS	One man among 26 patients with idiopathic NOA	NOA	Ghieh *et al.* (2022a)
*TDRD6*	c.1825G>T	p.Gly609X	Hom	Outside the Tudor domain	VUS	An infertile man with idiopathic OAT	OAT, round spermatid arrest	Bi *et al.* (2024)
	c.1259A>G	p.Tyr420Cys	Hom	Outside the Tudor domain	VUS	A 38-year-old patient from a consanguineous family	Oligozoospermia, OAT	Sha *et al.* (2018b)
*TDRD9*	c.1243G>T	p.Val415Phe	Hom	DEAD/DEAH-box helicase domain	VUS	39 infertile men	Extreme oligozoospermia or cryptozoospermia	Stallmeyer *et al.* (2024)
	c.3826G>T	p.Val1276Phe	Hom	Downstream of the Tudor domain				
*TEKT5*	c.531G>C	p.Glu177Asp	Het	Tektin domain	VUS	2 men among 37 men with MA and 194 men with SCOS	NOA, meiotic arrest, SCOS	Wyrwoll *et al.* (2022b)
	c.938C>G	p.Ser313Cys	Het	Tektin domain				
	c.1022C>T	p.Ala341Val	Het	Tektin domain				
*TEX14*	c.727C>G	p.Gln243Glu	Het	Pkinase domain	VUS	One patient among 16 patients with NOA from Ribeirao Preto, Brazil	NOA	Araujo *et al.* (2020)
	c.4297G>A	p.Glu1433Lys	Het	IDRs				
	c.4244A>C	p.Gln1415Pro	Hemi	IDRs	VUS	647 crypto-/azoospermic men	NOA	Wyrwoll *et al.* (2023)
*TEX33*	c.425T>G	p.Ile142Ser	Hom	IDRs	VUS	One man among 1056 crypto- and azoospermic individuals	Spermatogonial arrest	Sieper *et al.* (2024)
*TSPYL5*	c.1258C>T	p.Arg420Cys	Het	Outside the IDRs	VUS	1558 Han Chinese men with different spermatogenic conditions	Azoospermia	Yang *et al.* (2018)
*TULP2*	c.829C>A	p.Leu277Met	Het	TED consensus domain	VUS	300 unrelated infertile men	OAT	Zheng *et al.* (2021)
	c.832C>T	p.Arg278Trp	Het	TED consensus domain				
	c.871A>G	p.Thr291Ala	Het	TED consensus domain				
*WT1*	c.56T>A	p.Met19Lys	N.R.	Upstream of the ZnF domain	VUS	521 individuals presenting idiopathic primary SPGF	NOA or oligozoospermia	Lillepea *et al.* (2024)
	c.482T>A	p.Met161Lys		Upstream of the ZnF domain				
	c.542T>A	p.Met181Lys		Upstream of the ZnF domain				
	c.593T>A	p.Met198Lys		Upstream of the ZnF domain				
	c.1070T>A	p.Met357Lys		Classic ZnF domain				
	c.1121T>A	p.Met374Lys		Classic ZnF domain				
*WWC2*	c.617T>A	p.Met206Lys	Het	Downstream of the tryptophan-tryptophan WW2 domian	VUS	Patients with unexplained infertility	Severe SPGF	Hoffken *et al.* (2023)
	c.776T>C	p.Ile259Thr	Het	Downstream of the WW2 domian				
	c.1117C>T	p.Arg373Cys	Het	CCs				
	c.1184C>T	p.Ala395Val	Het	CCs				
	c.1192G>A	p.Glu398Lys	Het	CCs				
	c.1201_1206del	p.Arg401_Leu402	Het	CCs				
	c.2971C>A	p.His991Asn	Het	IDRs				
	c.3115G>A	p.Glu1039Lys	Het	Outside the IDRs and CCs				
**Teratozoospermia: Acrosome defects, ASS, and Globozoospermia**								
*BRDT*	c.2783G>A	p.Gly928Asp	Hom	Downstream of the bromo domain	VUS	A patient with acephalic spermatozoa	ASS	Li *et al.* (2017)
*HOOK1*	c.848T>C	p.Gln286Arg	Het	CCs	VUS	7 severe teratozoospermia patients	ASS	Chen *et al.* (2018)
*SPATA20*	c.619C>T	p.Arg207*	Hom	TED consensus domain	VUS	An ASS patient	ASS	Wang *et al.* (2023b)
**Asthenozoospermia: MMAF and PCD**								
*CCDC9*	c.232C>T	p.Arg78Trp	Hom	IDRs	VUS	26 individuals from rural Pakistani families	Absence of doublet microtubules and the central pair	Khan *et al.* (2025)
*CCDC40*	c.1450G>A	p.Glu484Lys	Het	Outside the CCs	VUS	Infertile patients with PCD	MMAF	Liu *et al.* (2021)
	c.2609G>A	p.Arg870His	Het	CCs				
*CYLC2*	c.551G>A	p.Gly184Asp	Het	IDRs	VUS	An infertile man of German origin	Abnormal morphology and absent acrosome	Schneider *et al.* (2023)
*DNAH3*	c.7477G>A	p.Asp2493Asn	Het	AAA 4 region	VUS	3 patients among 432 infertile Chinese men	Lost central pair of microtubules and dislocated mitochondrial sheath	Meng *et al.* (2024)
	c.8971C>T	p.Arg2991Cys	Het	Outside the CCs				
*DNAH17*	c.5408G>A	p.Cys1803Tyr	Hom	Outside the CCs	VUS	Three Pakistani infertile brothers	Asthenozoospermia	Zhang *et al.* (2020)
	c.10496C>T	p.Pro3499Leu	Het	AAA 5 region	VUS	5 infertile men	MMAF	Whitfield *et al.* (2019)
	c.612C>G	p.Ile204Met	Het	N-terminal region	VUS	An infertile patient and his family members		Song *et al.* (2023a)
	c.2150T>C	p.Leu717Pro	Het	N-terminal region				
	c.7136g>C	p.Trp2379Ser	Het	Outside the CCs				
	c.12865_12867delTCC	p.Ser4289del	Het	Downstream of the Tetratricopeptide repeat 3				
	c.13105C>T	p.Arg4369*	Het	Downstream of the Tetratricopeptide repeat 3				
*FSIP2*	c.5237AAG	p.Glu1747del	Het	Outside the IDRs and CCs	VUS	Four asthenoteratozoospermic patients	Flagellar defects, and acrosomal hypoplasia	Zheng *et al.* (2023)
	c.18448G>A	p.Val6150Ile	Het	Outside the IDRs and CCs				
	c.19981C>T	p.Arg6661Ter	Hom	Outside the IDRs and CCs				
*ODF2*	c.202A>G	p.Lys68Arg	Het	Outside the CCs	VUS	39 years old infertile patient	MMAF	Zhu *et al.* (2022b)

Gene variants from Clinvar and pubmed database. The domain data from Interpro database. AAA, ATPases Associated with diverse cellular Activities; ACMG, American College of Medical Genetics and Genomics; ASS, acephalic spermatozoa syndrome; BTB, Broad-Complex, Tramtrack and Bric a brac; CCs, coiled coils; Hom, Homozygous; Het, Heterozygous; Hemi, Hemizygous; IDRs, intrinsically disordered regions; MMAF, morphological abnormalities of the flagella; NOA, non-obstructive azoospermia; N.R., no reported. OAT, oligo-astheno-teratozoospermia; PCD, primary ciliary dyskinesia; Pkinase, protein kinase; POZ, poxvirus and zinc finger; RRM, RNA recognition motif; SCOS, Sertoli cell–only syndrome; SPGF, spermatogenic failure; TED, TRAF-EDD; VUS, variants of uncertain significance; WW, tryptophan-tryptophan; ZnF, zinc finger.

RBPs are indispensable for transcriptional and post-transcriptional RNA regulation, maintaining the stability, splicing, translation, and degradation of transcripts. These functions are essential for germ cell development, sperm morphology, and motility. Disruptions in RBP-encoding genes profoundly impact these processes, driving the diverse phenotypes observed in male infertility.

A recent study of 1046 infertile men identified a significant burden of RBP gene variants, including 22 loss-of-function variants in 18 genes and 137 damaging nonsynonymous variants in 105 genes (Li *et al.*, 2024c). Despite these findings, the majority of testis-expressed RBPs remain genetically and functionally uncharacterized.

To address this gap, we conducted a systematic screening of male infertility-associated variants across 1744 testis-expressed RBP genes using data from PubMed and ClinVar (Landrum *et al.*, 2018). In total, we identified 177 pathogenic variants in 62 RBP genes (Table 1) and 91 VUS in 35 RBP genes (Table 2). For each variant, we annotated the amino acid changes, zygosity information, affected protein domain—covering canonical RBDs (e.g. RRM, ZnF, DEAD/DEAH-box helicase), noncanonical RBDs (e.g. Tudor, Pkinase), and RNA-binding non-domain regions such as coiled coils—and its American College of Medical Genetics and Genomics (ACMG) classification, studied population, and mutant phenotypes.

**Figure 3.**
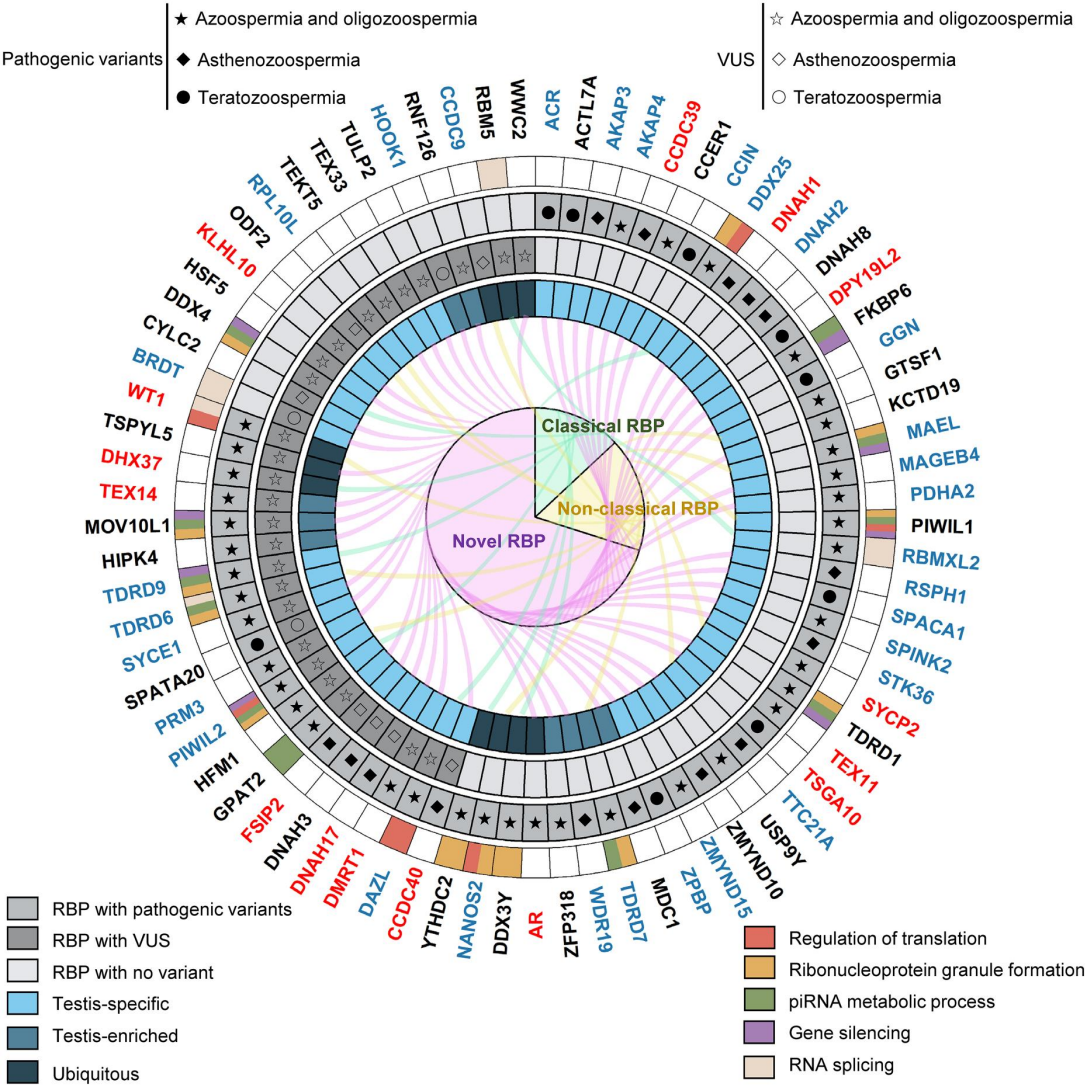
**Circos plot integrating functional annotation, genetic variation, tissue expression, and gene classification of RNA-binding proteins (RBPs) associated with male infertility.** The circos plot illustrates a comprehensive annotation of 177 pathogenic variants in 62 RBP genes and 91 variants of uncertain significance (VUS) in 35 RBP genes implicated in male infertility. From outer to inner layers: (i) The outermost circle displays five selected enriched gene ontology terms. Genes confidently linked to male infertility, as defined by the International Male Infertility Genomics Consortium, are labeled in red (moderate or higher classification); genes with limited or no supporting evidence are labeled in blue; and genes without sufficient data for classification are labeled in black. (ii) RBPs with pathogenic variants are shaded in dark gray, with solid shape symbols indicating disease types: azoospermia/oligozoospermia (★), asthenozoospermia (◆), teratozoospermia (●). (iii) RBPs with VUS are also marked in dark gray, with hollow symbols denoting the same disease types: azoospermia/oligozoospermia (☆), asthenozoospermia (◇), teratozoospermia (○). (iv) Tissue specificity is shown in blue: testis-specific (light), testis-enriched (medium), ubiquitous (dark). (v) The inner pie chart classifies RBPs into three categories: classical (green), non-classical (yellow), and novel (purple); segment size reflects gene count. (vi) Radial lines link individual RBPs to their classification group and are colored accordingly. This integrative visualization facilitates functional and clinical prioritization of candidate RBPs in male infertility. piRNA, piwi-interacting RNA.

Among these, 15 were classified as having moderate to definitive evidence of association with male infertility by IMIGC database (Houston *et al.*, 2021). These genes are thus considered confidently linked to human male infertility. An additional 31 genes demonstrated limited or no evidence, while the remaining 31 genes lack sufficient data for classification. Based on domain structure, these RBPs comprise 10 classical, 13 non-classical, and 54 novel types. According to testis expression profiles, 58 are testis-specific, 9 are enriched, and 10 are ubiquitously expressed (Fig. 3).

To further explore their potential functions, we performed Gene Ontology (GO) and Kyoto Encyclopedia of Genes and Genomes (KEGG) pathway enrichment analyses. *DDX25, PIWIL1, NANOS2, DAZL, WT1*, and *PIWIL2* were enriched in pathways related to the regulation of translation. Several genes, including *FKBP6, MAEL, PIWIL1, PIWIL2, GPAT2, TDRD1, TDRD6, TDRD7, TDRD9, MOV10L1*, and *DDX4*, participated in the piRNA metabolic process. *RBMXL2, TDRD6, WT1, BRDT*, and *RBM5* were implicated in RNA splicing. In addition, certain genes were enriched in processes such as ribonucleoprotein (RNP) granule formation and gene silencing.

Collectively, these findings underscore the current advancements in elucidating the genetic underpinnings of male infertility and provide a comprehensive framework for future investigations. Further validation of these candidate RBPs may reveal novel genetic contributors to male reproductive health and offer new targets for diagnostic and therapeutic strategies.

### RBPs in azoospermia and oligozoospermia: regulating germ cell proliferation and meiosis

Azoospermia and oligozoospermia, characterized by the absence or significant reduction of spermatozoa, often arise from disruptions in germ cell proliferation, meiotic progression, and differentiation. These processes are critically dependent on RBPs, which regulate post-transcriptional mechanisms such as RNA stability, splicing, and homeostasis to ensure precise gene expression during spermatogenesis. Mutations in key RBP genes disrupt these tightly controlled pathways, impairing fundamental mechanisms such as piRNA biogenesis, RNA helicase activity, and chromosomal stability. Such dysfunctions, occurring at any stage of spermatogenesis—from the self-renewal of spermatogonia to the formation of haploid spermatids—compromise male fertility by disrupting the molecular events required for germ cell development and genomic integrity.

A cornerstone of genomic stability in germ cells is the piRNA pathway, which is intricately regulated by the TDRD family among RBPs. Mutations in *TDRD1* (Stallmeyer *et al.*, 2024), such as c.887C>A identified in 39 men with non-obstructive azoospermia (NOA), impair RNA granule formation and lead to meiotic arrest. Similarly, *TDRD7* (Tan *et al.*, 2019), which stabilizes and transports meiotic transcripts, exhibits mutations like c.324_325insA in 176 NOA patients, disrupting spermatogonial differentiation. Further supporting these processes is *TDRD9* (Wang *et al.*, 2024b), whose mutations (e.g. c.958delC) found in Chinese oligozoospermic families underscore its critical role in balancing spermatogonial self-renewal and differentiation.

Beyond piRNA, RNA helicases in the DDX family play essential roles in maintaining RNA structural integrity during translation and splicing. For instance, mutations in *DDX3Y* (e.g. c.1230_1231del) (Dicke *et al.*, 2023; Subrini *et al.*, 2025), identified in 1655 infertile men, impair helicase activity required for meiotic progression, frequently resulting in Sertoli cell-only syndrome (SCOS). Meanwhile, *DDX25* mutations (Kherraf *et al.*, 2022), such as c.1129C>T reported in North African patients, destabilize mRNA translation, preventing the transition from spermatogonia to spermatocytes.

Chromosomal stability during meiosis represents another critical layer of regulation by RBPs. Mutations in HFM1 (Tang *et al.*, 2021), such as c.3490C>T observed in 2 of 51 patients with NOA, result in meiotic arrest at the metaphase stage. Similarly, mutations in synaptonemal complex proteins such as *SYCP2* (c.3067_3071del) (Schilit *et al.*, 2020) and *SYCE1* (c.689_690del) (Feng *et al.*, 2022a) destabilize chromosome pairing and crossover formation, further compromising meiotic progression.

RBPs also integrate transcriptional and translational control mechanisms to regulate RNA splicing and stability, both of which are essential for germ cell development. *RBMXL2* (Ghieh *et al.*, 2022b), a critical splicing regulator, ensures the production of meiosis-specific isoforms; its mutations c.301dup lead to defective spermatogenesis. Downstream, *DAZ*L (Li *et al.*, 2024c), a master regulator of meiotic mRNA translation, protects essential transcripts required for germline development. Disruptions in piRNA processing by *MOV10L1* mutations (c.3094_3097del) (Li *et al.*, 2022a) further highlight the interconnectedness of RBP functions, as these mutations lead to transcript degradation, genomic instability, and germ cell apoptosis.

In summary, RBPs govern multiple interconnected pathways during spermatogenesis, and their dysfunction underlies the pathophysiology of azoospermia and oligozoospermia. These insights highlight the indispensable roles of RBPs in germ cell proliferation and meiosis.

### RBPs in teratozoospermia: coordinating morphogenesis and structural integrity

Teratozoospermia, marked by abnormal sperm morphology, arises from defects in key processes such as acrosome formation, cytoskeletal organization, and nuclear condensation. RBPs are indispensable in regulating these tightly orchestrated events, ensuring proper protein trafficking, vesicle fusion, and chromatin remodeling. Disruptions in RBP-associated pathways compromise the structural and functional integrity of spermatozoa, resulting in impaired fertility.

Acrosome biogenesis, a critical step in sperm development, requires the precise transport and fusion of protein-containing vesicles to form a functional acrosome. Mutations in *DPY19L2* (Celse *et al.*, 2021), such as c.892C>T identified in 69 globozoospermic patients, disrupt the anchoring of the acrosome to the nuclear membrane, resulting in round-headed sperm that lack the ability to penetrate the egg. Similarly, *ACTL7A* (Xin *et al.*, 2020; Wang *et al.*, 2021), which organizes actin filaments essential for vesicle transport, is affected by mutations like c.733G>A, leading to incomplete acrosomal formation.

Mutations in *ZPBP* (e.g. c.931C>T) (Oud *et al.*, 2020) and *SPACA1* (e.g. c.53G>A) (Chen *et al.*, 2021b) further impair vesicle fusion, preventing the proper assembly of the acrosome and contributing to structural abnormalities. These findings illustrate how RBPs coordinate vesicle dynamics and fusion, ensuring the correct localization and assembly of acrosomal components during spermiogenesis.

Cytoskeletal integrity, vital for nuclear shaping and head-tail attachment, is another key process regulated by RBPs. During spermatid elongation, RBPs such as *HOOK1* guide the formation of the manchette, a transient microtubule structure that determines the alignment and connection between the sperm head and tail. Mutations in *HOOK1* (Chen *et al.*, 2018), such as c.848T>C, maybe disrupt manchette formation, resulting in acephalic spermatozoa syndrome (ASS), a condition characterized by decapitated sperm. Similarly, *TSGA10* (Sha *et al.*, 2018a), a regulator of chromatin remodeling during nuclear shaping, is frequently mutated in teratozoospermic individuals, with variants such as c.211del reported in Turkish families. These mutations disrupt chromatin condensation, exacerbating structural abnormalities and impairing sperm function.

Nuclear condensation, the hallmark of sperm maturation, is tightly regulated by RBPs that mediate the transition from histones to protamines. This process ensures compact DNA packaging and genomic stability, which are essential for fertilization. *TDRD7* plays a crucial role in stabilizing transcripts that encode chromatin remodeling enzymes, facilitating the histone-to-protamine (HTP) transition. Disruptions in *TDRD7* (Wang *et al.*, 2024b) function result in poorly condensed nuclei and compromised DNA integrity.

RBPs function at the intersection of multiple critical pathways, coordinating acrosome formation, cytoskeletal organization, and nuclear remodeling to ensure the structural and functional integrity of spermatozoa. Mutations in key genes such as *DPY19L2*, *TSGA10*, and *TDRD7* highlight the interconnected nature of these processes and their essential roles in sperm morphogenesis. Deciphering RBP-regulated pathways provides key insights into teratozoospermia and potential targets for treating sperm morphology–related male infertility.

### RBPs in asthenozoospermia: sustaining flagellar function and energy production

Asthenozoospermia, characterized by reduced sperm motility, arises from impairments in flagellar structure, radial spoke integrity, and energy metabolism (Eisenberg *et al.*, 2023). RBPs critically regulate transcripts encoding axonemal and mitochondrial proteins, thereby controlling flagellar assembly, stability, and function. Disruptions in these tightly coordinated pathways lead to immotile or dysfunctional sperm, a hallmark of male infertility.

The structural integrity of the flagellum, which is essential for sperm motility, depends on the precise assembly and stabilization of axonemal components, particularly dynein arms that generate the sliding motion of microtubules. *DNAH1* (Yu *et al.*, 2021), a gene encoding an axonemal dynein heavy chain, is crucial for the function of outer dynein arms. Mutations such as c.11726_11727del, identified in 41 Chinese patients with multiple morphological abnormalities of the flagella (MMAF), result in defective dynein arm assembly, rendering sperm immotile. Similarly, mutations in DNAH8 (e.g. c.6158_6159insT) (Dil *et al.*, 2023) and *DNAH17* (c.1293_1294del) (Whitfield *et al.*, 2019; Song *et al.*, 2023a), reported in Pakistani and French MMAF cohorts, highlight the conserved role of dynein-related genes in maintaining axonemal function and motility. These findings underscore the critical role of RBPs in stabilizing and translating transcripts required for dynein arm integrity during flagellar development.

Radial spokes, essential for converting dynein-generated sliding into coordinated flagellar bending, rely on RBPs to regulate their assembly and maintenance. Mutations in *CCDC39* (e.g. c.1072del) (Aprea *et al.*, 2023) and *CCDC40* (e.g. c.901C>T) (Liu *et al.*, 2021) destabilize the radial spoke structure, leading to axonemal disorganization and loss of motility, as observed in primary ciliary dyskinesia (PCD). Similarly, mutations in *RSPH1* (e.g. c.680dup) (Aprea *et al.*, 2023) impair radial spoke assembly, compounding motility defects. By coordinating the expression and localization of radial spoke components, RBPs ensure the structural integrity of the axoneme, which is essential for efficient flagellar motion.

Energy production, another critical component of sperm motility, is closely linked to mitochondrial function, which is also regulated by RBPs. The mitochondrial sheath, a structure surrounding the midpiece of the sperm, provides the ATP for flagellar movement. Mutations in *WDR19* (e.g. c. A3811G) (Ni *et al.*, 2020) disrupt the formation of this sheath, leading to reduced ATP production and impaired motility. Similarly, mutations in *MDC1* (e.g. c.472C>T) (Oud *et al.*, 2021) highlight the role of RBPs in stabilizing transcripts involved in oxidative phosphorylation, further underscoring their importance in maintaining mitochondrial function and energy homeostasis.

RBPs integrate these processes to coordinate the structural and metabolic demands of sperm motility. By regulating axonemal assembly, radial spoke stability, and mitochondrial energy production, RBPs ensure the functionality of the flagellum, which is indispensable for male fertility. Mutations in key genes such as *DNAH1* (Amiri-Yekta *et al.*, 2016; Yu *et al.*, 2021), *RSPH1* (Aprea *et al.*, 2023), and *WDR19* (Ni *et al.*, 2020) exemplify the interconnected nature of these pathways, providing valuable insights into the molecular mechanisms underlying asthenozoospermia. These findings not only deepen our understanding of RBP-mediated regulation in sperm motility but also offer potential therapeutic targets for addressing male infertility caused by motility defects.

Additionally, VUS in RBP genes have been identified in individuals with male infertility phenotypes, including azoospermia, oligozoospermia, teratozoospermia, and asthenozoospermia (Table 2). While these variants suggest potential links to disrupted spermatogenesis, their pathogenicity remains unverified. For instance, VUS in *DDX4*, *DMRT1*, and *HFM1* have been observed in patients with NOA or oligozoospermia, raising the possibility of their involvement in spermatogonial maintenance, RNA stability, and meiotic progression.

Similarly, variants in BRDT (Li *et al.*, 2017) and SPATA20 (Martinez *et al.*, 2023) have been associated with teratozoospermia, hinting at roles in acrosome formation and cytoskeletal organization. In asthenozoospermia, VUS in genes such as ODF2 (Zhu *et al.*, 2022b), CYLC2 (Schneider *et al.*, 2023), and CCDC9 (Khan *et al.*, 2025) have been reported, potentially implicating them in axonemal assembly and flagellar function. However, the precise contributions of these variants to infertility remain speculative and require rigorous functional validation. Future research should prioritize elucidating the molecular mechanisms underlying these VUS through advanced experimental approaches. Integrating genomic data with detailed clinical phenotypes will be essential to determine their diagnostic value and therapeutic potential, ultimately advancing the understanding and management of male infertility.

## Roles of RBPs in mouse spermatogenesis

Spermatogenesis, the complex process through which male germ cells develop into mature spermatozoa, is divided into three sequential stages: spermatocytogenesis (proliferation and differentiation of spermatogonia), spermatidogenesis (meiosis of spermatocytes), and spermiogenesis (differentiation of round spermatids) (Jordan *et al.*, 1961; Wang *et al.*, 2022a). Each stage involves tightly regulated transcriptional and post-transcriptional programs mediated by RBPs.

**Figure 4.**
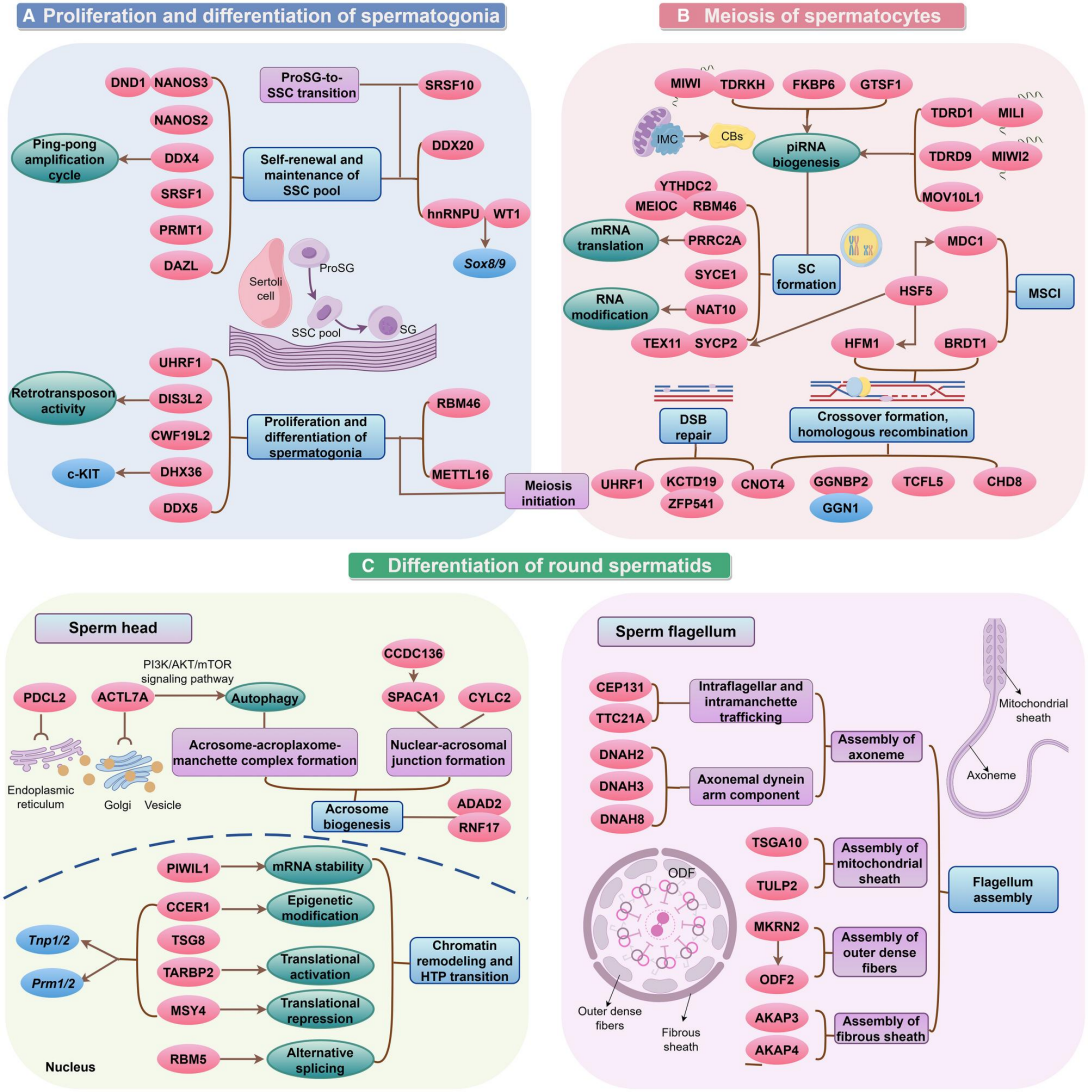
**Regulatory roles of RNA-binding proteins (RBPs) across different stages during spermatogenesis in mice.** This diagram summarizes the stage-specific regulatory functions of RBPs during mouse spermatogenesis, from spermatocytogenesis to spermiogenesis. (**A) Proliferation and differentiation of spermatogonia**: RBPs such as NANOS3, NANOS2, DDX4, and others are essential for the self-renewal and maintenance of spermatogonial stem cells. The transition to prospermatogonia is tightly controlled by RBPs including hnRNPU, DDX20, and SRSF10. Notably, hnRNPU interacts with WT1 to regulate transcription factors *Sox8*/*9*. In addition, DIS3L2, DHX36, RBM46, METTL16, and others are involved in promoting spermatogonial proliferation and differentiation, with RBM46 and METTL16 also facilitating the initiation of meiosis. (**B) Meiosis of spermatocytes**: During meiosis, the biogenesis of piwi-interacting RNAs is tightly regulated by several RBPs, such as TDRKH/MIWI, TDRD1/MILI, TDRD9/MIWI2, and others. RBPs also modulate critical chromosomal events such as synaptonemal complex formation, DNA double-strand break repair, crossover formation, homologous recombination, and meiotic sex chromosome inactivation (MSCI). Among these regulators, HSF5 directly binds to the promoters of key genes—*Hfm1* (crossover formation), *Sycp2* (synapsis), and *Mdc1* (MSCI)—and controls their transcription during meiotic progression. (**C) Differentiation of round spermatids**: In post-meiotic stages, RBPs coordinate essential processes including chromatin remodeling, the histone-to-protamine transition, acrosome biogenesis, and flagellar assembly. RBPs such as CCER1, PIWIL1, MSY4, and others regulate chromatin remodeling through post-transcriptional control of spermatid mRNAs. ACTL7A participates in the PI3K/AKT/mTOR pathway to maintain the integrity of the acrosome-acroplaxome-manchette complex and suppress autophagy. Furthermore, RBPs like CEP131, ODF2, members of the DNAH, AKAP family, and others are essential for flagellum assembly and structural integrity. Collectively, this model underscores the multifaceted and tightly coordinated roles of RBPs in regulating key molecular events throughout spermatogenesis, highlighting their indispensable contributions to male germ cell development and fertility. CBs, chromatoid body; DSB, DNA double-strand break; HTP, histone-to-protamine; IMC, intermitochondrial cement; ODF, outer dense fibers; piRNA, piwi-interacting RNA; ProSG, prospermatogonia; SC, synaptonemal complex; SG, spermatogonia; SSCs, spermatogonial stem cells.

Building on recent findings published in *Science*, which identified 1744 RBPs in mouse testes, we conducted an in-depth analysis using the PubMed database to identify 124 RBP gene knockout mice associated with male infertility. This section synthesizes the roles of these RBPs across the different stages of spermatogenesis, emphasizing their molecular regulatory mechanisms and germline-specific contributions (Fig. 4). Additionally, we provide a comprehensive summary of these knockout models and their associated phenotypes in Table 3, detailing the molecular pathways disrupted by RBP dysfunction and their implications for male fertility.

**Table 3.** Phenotypes and regulatory mechanisms in RNA-binding protein gene knockout/mutant male mice.

Genes	KO/MUT models	Fertility	Phenotypes	Regulatory mechanisms	Ref.
**Proliferation and differentiation of spermatogonia**					
*Cwf19l2*	cKO in GCs	Infertile	Increased apoptosis and decreased differentiated spermatogonia	Regulate AS during the early steps of spermatogenesis	Wang *et al.* (2024a)
*Dazl*	cKO in GCs	Infertile	Dramatically fewer differentiating spermatogonia	Regulate Pro-SG expansion and differentiation	Mikedis *et al.* (2020)
*Ddx4 (Mvh)*	KO mice	Infertile	Defective SSC maintenance	Regulate piRNA processing and gene silencing of retrotransposons	Kuramochi-Miyagawa *et al.* (2010)
*Ddx5*	cKO in GCs	Infertile	Rapid and profound depletion of spermatogonia	Regulate gene expression programs and activity of undifferentiated spermatogonia	Legrand *et al.* (2019)
*Ddx20*	cKO in GCs	Infertile	Neither SSC pool nor Pro-SG formed	Regulate cell-cycle reentry of Pro-SG and establishment of SSC pool	Zou *et al.* (2024)
*Dis3l2*	cKO in GCs	Infertile	Impaired differentiation of spermatogonia	Safeguard the correct transcriptome during spermatogonial differentiation	Li *et al.* (2024b)
*Dhx36*	cKO in GCs	Infertile	Decreased spermatogonial differentiation	Regulate spermatogonia differentiation by promoting c-kit expression	Gao *et al.* (2015)
*Hnrnpu*	cKO in Pro-SG	Infertile	Accumulated Pro-SG and disrupted process of T1-ProSG to T2-ProSG transition	Participate in SSC pool establishment	Wen *et al.* (2024b)
	cKO in SCs	Infertile	Failed development and migration of spermatogonia	Cooperate with WT1 in SCs and modulate transcriptional factors *Sox8/9*	Wen *et al.* (2021)
*Hnrnpa2b1*	KO mice	Infertile	Wholly depleted GCs at post-natal day 7	Regulate stress granules disassembly	Wang *et al.* (2024b)
*Mettl16*	cKO in GCs	Infertile	Impaired spermatogonia differentiation	Control the mitosis-to-meiosis GC fate decision	Ma *et al.* (2024)
*Nanos2*	cKO in GCs	Infertile	Gradually lost SSCs and completely disappeared GCs	Self-renew SSCs and maintain the stem cell state	Sada *et al.* (2009)
*Nanos3*	KO mice	Infertile	Gradually lost SSCs and completely absent GCs	Cooperate with DND1 to suppress the entry of germ cell lineage	Tsuda *et al.* (2003), Wang *et al.* (2025)
*Prmt1*	cKO in GCs	Infertile	Impaired spermatogonial establishment and maintenance	Coordinate the germline arginine methylome	Azhar *et al.* (2023)
*Rbm46*	KO mice	Infertile	Impaired differentiation of spermatogonia	Regulate spermatogonia differentiation prior to the commitment to meiosis	Peart *et al.* (2022)
*Uhrf1*	cKO in Pro-SG	Infertile	Sertoli cell-only syndrome phenotype	Regulate AS during SSC homeostasis	Zhou *et al.* (2022)
*Upf2*	cKO in SCs	Infertile	Failed migration of spermatogonia to the basal compartment	Regulate nonsense-mediated mRNA decay	Bao *et al.* (2015)
*Wt1*	cKO in GCs	Infertile	Accumulated undifferentiated spermatogonia and disrupted BTB	Regulate the polarity of SCs via Wnt signaling pathway	Zheng *et al.* (2014)
*Srsf1*	cKO in GCs	Infertile	Absent GCs and impaired homing of precursor SSCs	Regulate AS and play a role in spermatogonia survival	Lei *et al.* (2023b), Sun *et al.* (2024)
*Srsf2*	cKO in GCs	Infertile	Defective spermatogonial differentiation	Regulate AS	Lei *et al.* (2023a)
*Srsf10*	cKO in GCs	Infertile	Impaired differentiation of spermatogonia	Regulate AS to maintain the expansion of undifferentiated Pro-SG	Liu *et al.* (2022a)
*Ythdf2*	KO mice	Infertile	Impaired spermatogonial proliferation and affected G2/M transition	Modulate matrix metallopeptidase decay via m6A/mRNA pathway	Huang *et al.* (2020)
**Meiosis of spermatocytes**					
*Alkbh5*	KO mice	Infertile	Delayed meiotic progress	Mediate m6A eraser in the nuclei of spermatocytes and maintain BTB integrity	Cai *et al.* (2022), Tang *et al.* (2018)
*Ar*	cKO in SCs	Infertile	Arrested meiosis at the diplotene stage	Regulate androgen maintenance of complete spermatogenesis	Chang *et al.* (2004)
*Brdt*	Mutant mice	Infertile	Blocked meiosis I is and completely absent GCs	Regulate proper chromatin organization, silencing of sex chromosomes and crossover formation	Manterola *et al.* (2018)
*Cnot4*	cKO in GCs	Infertile	Defective DNA damage repair and homologous crossover between X and Y chromosomes	Regulate mRNA Degradation, efficient DNA damage repair, and XY chromosome crossover	Dai *et al.* (2021)
*Cstf2t*	KO mice	Infertile	Defects in chromosomal condensation	Interact with pre-mRNAs at sequences downstream of the cleavage site	Grozdanov *et al.* (2018)
*Dazl*	cKO in GCs	Infertile	Arrested meiosis	Participate in translational regulation	Li *et al.* (2019a)
*Dazap1*	KO mice	Infertile	Arrested meiosis and increased GC apoptosis	Regulate mRNA transcription, splice and translation	Sasaki *et al.* (2018)
*D1pas1*	KO mice	Infertile	Arrested meiosis in late pachytene spermatocytes	Resolve R-loops and form a complex for mouse pachytene piRNA biogenesis	Choi *et al.* (2024)
*Dis3l2*	cKO in GCs	Infertile	Blocked meiosis, and increased apoptosis of spermatocytes	Safeguard the correct transcriptome during spermatocyte meiotic progression	Li *et al.* (2024b)
*Fkbp6*	KO mice	Infertile	Abnormal pairing and misalignments between homologous chromosomes	Regulate piRNA amplification and transposon silencing	Crackower *et al.* (2003), Wyrwoll *et al.* (2022a)
*Fus (Tls)*	KO mice	Infertile	Blocked meiosis I at pachytene stage	Play a role in homologous DNA pairing and recombination	Kuroda *et al.* (2000)
*Gtsf1*	KO mice	Infertile	Blocked Meiotic progression before the zygotene stage	Regulate retrotransposon suppression	Yoshimura *et al.* (2009)
*Ggnbp2*	KO mice	Infertile	Compromised DSB repair and reduced crossovers in spermatocytes	Interact with GGN1 to regulate DSB repair	Guo *et al.* (2018)
*Hadh*	KO mice	Infertile	Blockage of meiosis in spermatocytes	Regulate TNF-α/Bcl-2 signaling pathway	Zhu *et al.* (2022a)
*Hsf5*	KO mice	Infertile	Blocked meiosis I at prophase	Modulate the transcriptome to ensure meiotic progression	Liu *et al.* (2024a), Luo *et al.* (2024)
*Hfm1*	KO mice	Infertile	Meiotic block with crossover formation defects	Required for crossover formation and complete synapsis of homologous chromosomes	Guiraldelli *et al.* (2013)
*Hnrnph1*	cKO in SCs	Infertile	Delayed meiosis and damaged BTB function	Recruit PTBP2 and SRSF3 to modulate AS and establish SC-GC crosstalk through cooperation with PTBP1 and AR	Feng *et al.* (2022b), Feng *et al.* (2023)
*Hnrnpk*	cKO in GCs	Infertile	Blocked Meiosis I at pachytene stages	Regulate the expression of genes involved in meiosis	Xu *et al.* (2022a)
*Kctd19*	KO mice	Infertile	Arrested meiosis I at prophase	Form ZFP541/KCTD19 signaling complex to regulate pachytene progression	Li *et al*. (2022b)
*Larp7*	cKO in GCs	Infertile	Blocked meiosis	Mediate U6 snRNA 2′-O-methylation to ensure splicing fidelity	Wang *et al.* (2020b)
*Mael*	KO mice	Infertile	Massive DNA damage and severe chromosome asynapsis during meiosis	Regulate transposon repression	Soper *et al.* (2008)
*Mdc1*	KO mice	Infertile	Meiotic arrest	Direct chromosome-wide silencing of the sex chromosomes in male GCs	Ichijima *et al.* (2011)
*Meioc*	KO mice	Infertile	Blocked meiosis I and completely absent GCs at post-meiotic phase	Participate in the post-transcriptional regulation of target transcripts	Soh *et al.* (2017)
*Mili*	KO mice	Infertile	Blocked meiosis I at early prophase	Regulate piRNA clusters in transposon control	Aravin *et al.* (2007)
*Mov10l1*	KO mice	Infertile	Blocked meiosis I at prophase	Interact with PLD6 to regulate piRNA biogenesis	Guan *et al.* (2021), Zheng *et al.* (2010)
*Nat10*	cKO in GCs	Infertile	Severely inhibited meiotic entry	Mediate N4-acetylcytidine modification	Chen *et al.* (2022)
*Prrc2a*	cKO in GCs	Infertile	XY asynapsis and impaired meiotic sex chromosome inactivation and delayed metaphase entry	Act as a reader protein to exert m6A regulation mechanisms	Tan *et al.* (2023b)
*Rbm46*	cKO in GCs	Infertile	Blocked Meiosis I	Down-regulate mitotic transcripts during meiosis entry	Qian *et al.* (2022)
*Rpl10l*	KO mice	Infertile	Delayed prophase-to-metaphase transition during meiosis I	Compensate for RPL10 during Meiotic Sex Chromosome Inactivation	Jiang *et al.* (2017)
*Setx*	KO mice	Infertile	Blocked Meiosis I at pachytene stage	Regulate meiotic recombination and gene silencing	Wen *et al.* (2024a)
*Scaper*	KO mice	Infertile	Completely absent spermatocytes	Modulate microtubule dynamics during meiosis	Wormser *et al.* (2021)
*Sam68*	KO mice	Infertile	Defective meiosis and increased GC apoptosis	Regulate mRNA translation	Paronetto *et al.* (2009)
*Sycp2*	Mutant mice	Infertile	Defective chromosome synapsis during meiosis	Regulate synaptonemal complex assembly and chromosomal synapsis	Yang *et al.* (2006)
*Syce1*	KO mice	Infertile	Disrupted meiosis and lacked spermatid	Interact with RAD51 to promote homologous synapsis from sites of recombination	Bolcun-Filas *et al.* (2009)
*Tex14*	Mutant mice	Infertile	Blocked meiosis	Regulate the conversion of midbodies into intercellular bridges	Greenbaum *et al.* (2006)
*Tdrd1*	KO mice	Infertile	Arrested meiosis at leptotene or zygotene stage	Drive intermitochondrial cement assembly to promote piRNA biogenesis	Gao *et al.* (2024)
*Tdrkh (Tdrd2)*	cKO in GCs	Infertile	Defective Meiotic division	Recruit MIWI to engage the piRNA pathway	Ding *et al.* (2019), Saxe *et al.* (2013)
*Tdrd9*	KO mice	Infertile	Blocked meiosis	Silence LINE1 retrotransposons	Wenda *et al.* (2017)
*Tcfl5*	KO mice	Infertile	Arrested meiosis	Regulate the transcription of spermatogenesis-related genes	Galan-Martinez *et al.* (2022)
*Tex11*	Mutant mice	Infertile	Impaired meiosis	Involved in initiation and/or maintenance of chromosome synapsis and formation of crossovers	Yang *et al.* (2015)
*Uhrf1*	cKO in GCs	Infertile	Failed meiosis	Involve in epigenetic regulation by suppressing retrotransposons and cooperating with PRMT5 and PIWI proteins in male germ cells	Dong *et al.* (2019), Pan *et al.* (2020)
*Wt1*	cKO in GCs	Infertile	Arrested meiosis and disrupted BTB	Regulate the polarity of SCs via Wnt signaling pathway	Zheng *et al.* (2014)
*Wnk1*	cKO in GCs	Infertile	Arrested mid-pachytene spermatocyte differentiation	Regulate mTOR activity and impact translation on a broader spectrum	Chi *et al.* (2023)
*Wdr62*	cKO in GCs	Infertile	Prolonged meiosis I at the metaphase stage	Involved in centriole duplication	Ho *et al.* (2021)
*Ythdc2*	KO mice	Infertile	Arrested meiosis at the zygotene stage	Modulate the levels of m6A-modified germline transcripts	Hsu *et al.* (2017), Wojtas *et al.* (2017)
*Zfp541*	KO mice	Infertile	Arrested meiosis at the diplotene stage	Form ZFP541/KCTD19 signaling complex to regulate pachytene progression	Li *et al.* (2022b), Xu *et al.* (2022b)
**Differentiation of spermatids**					
*Adad1*	KO mice	Infertile	Defective DNA compaction, abnormal head shaping, and reduced motility	Regulate normal translation of nuclear pore and transport protein transcripts	Potgieter *et al.* (2023)
*Adad2*	KO mice	Infertile	Arrested round spermatid differentiation and abnormal acrosome formation	Interact with RNF17 to repress ping-pong cycle in piRNA biogenesis	Xiong *et al.* (2023)
*Actl7a*	KO mice	Infertile	Abnormal shape of sperm heads	Regulate the formation of acrosome-acroplaxome-manchette complex	Zhang *et al.* (2023)
*Actl7b*	KO mice	Infertile	Multiple morphological abnormalities of the flagellum	Regulate spermatid morphogenesis	Clement *et al.* (2023)
*Akap3*	KO mice	Infertile	Morphological abnormalities of the spermatids	Regulate the formation of the specific subcellular structure of the sperm flagellum and proteome	Xu *et al.* (2020)
*Akap4*	KO mice	Infertile	Abnormal sperm morphology and motility	Participate in the composition of sperm fibrous sheath	Fang *et al.* (2019)
*Bsg*	KO mice	Infertile	Blocked round spermatid differentiation and compromised BTB integrity	Regulate Sertoli-germ cell crosstalk	Bi *et al.* (2013)
*Bscl2*	KO mice	Infertile	Increased chromocenter fragmentation and disrupted acrosome formation	Regulate spermatid chromatin integrity, acrosome formation, and mitochondrial activity	El Zowalaty *et al.* (2015)
*Boll (Boule)*	KO mice	Infertile	Blocked differentiation of round spermatids	May act by binding to the mRNAs and regulating their translation	VanGompel and Xu (2010)
*Ccdc136*	KO mice	Infertile	Severe disrupted acrosome formation	Regulate the expression levels of proteins (SPACA1 and PICK1)	Geng *et al.* (2016)
*Ccer1*	KO mice	Infertile	Defective sperm chromatin compaction	Coordinate the histone epigenetic modifications, histone-to-protamine transitions, chromatin condensation	Qin *et al.* (2023)
*Ccin*	Mutant mice	Infertile	Severe head malformation of spermatozoa	Required for both nuclear and acrosomal shaping	Fan *et al.* (2022)
*Cdkn2aip*	KO mice	infertile	Defective sperm heads	May regulate protamine replacement	Cao *et al.* (2022)
*Celf1*	KO mice	Infertile	Arrested differentiation of round spermatids	Regulate the translation of *Cyp19a1* mRNAs	Boulanger *et al.* (2015), Cibois *et al.* (2012)
*Cep131*	Mutant mice	Infertile	Defective flagella of sperm	Regulate the cilium/flagellum formation	Hall *et al.* (2013)
*Cul4b*	cKO in GCs	Infertile	Impaired sperm mobility	Regulate ubiquitin modification during spermatogenesis	Yin *et al.* (2016)
*Cylc2*	KO mice	Infertile	Morphological defects of the sperm head, acrosome and midpiece	Regulate the formation of acrosome	Schneider *et al.* (2023)
*Cstf2t*	KO mice	Infertile	Disrupted post-meiotic development, and oligoasthenoteratozoospermia	Regulate expression of histones and histone-like proteins	Grozdanov *et al.*, (2018)
*Dazl*	cKO in GCs	Infertile	Arrested differentiation of round spermatids	Participate in translational regulation	Li *et al.* (2019a)
*Ddx43*	KO mice	Infertile	Defective histone-to-protamine replacement and post-meiotic chromatin condensation	Regulate dynamic RNA regulatory processes	Tan *et al.* (2023a)
*Ddx25*	KO mice	Infertile	Round spermatids arrest at step 8 and failed elongation	Participate in mRNA export and translation regulation	Raju *et al.*, (2021)
*Dnah1*	KO mice	Infertile	Asthenozoospermia and reduced ciliary beat	Regulate the function of cilia and sperm flagella	Zhuang *et al.* (2022)
*Dnah2*	Mutant mice	Infertile	Morphological Abnormalities of the Sperm Flagella phenotype	Regulate sperm flagella formation	Hwang *et al.* (2021)
*Dnah3*	KO mice	Infertile	Abnormal sperm flagella of sperm	May regulate sperm mitochondrial sheath formation	Meng *et al.* (2024)
*Dnah8*	KO mice	Infertile	Abnormal sperm flagella and diminished sperm movement	Force generating protein component of the outer dynein arms in the sperm flagellum	Liu *et al.* (2020)
*Dpy19l2*	KO mice	Infertile	Disrupted vesicular trafficking, failed sperm nuclear shaping and eliminated unbound acrosomal vesicle	Regulate sperm head elongation and acrosome formation	Pierre *et al.* (2012)
*Ewsr1*	cKO in GCs	Infertile	Impaired spermatid maturation and chromosome centromere formation	Play an important role in regulation of spermiogenesis-related mRNA synthesis	Tian and Petkov (2021)
*Fxr1*	cKO in GCs	Infertile	Arrested differentiation of the round spermatids	Activate the translation of stored mRNAs	Kang *et al.* (2022)
*Gykl1*	KO mice	Infertile	Dysfunctional spermatozoa and defective sperm tail	Cooperate with *Pld6* in regulating sperm mitochondrial sheath formation	Chen *et al.* (2017)
*Gpx4*	cKO in GCs	Infertile	Severe abnormalities in spermatozoa	Maintain the normal structure of flagella and mitochondria in the spermatozoa	Imai *et al.* (2009)
*Henmt1*	Mutant mice	Infertile	Spermiogenesis arrest	Mediate the methylation of the 3′ end of piRNAs	Li *et al.* (2024a)
*Hnrnpc*	cKO in SCs	Infertile	Defective spermiogenesis and impaired BTB	Maintain the function of SCs and sustain steady-state spermatogenesis	Mo *et al.* (2024)
*Hspa4l*	KO mice	Infertile	Malformed sperm heads	May regulate the posttranslational modification of histones	Itoh *et al.* (2019)
*Hsp90b1*	cKO in GCs	Infertile	Large and globular heads with abnormal intermediate pieces	Regulate the transport of proteins in the endoplasmic reticulum	Audouard and Christians (2011)
*Klhl10*	Mutant mice	Infertile	Asynchronous spermatid maturation	Mediate the ubiquitination and subsequent proteasomal degradation of target proteins	Giassetti *et al.* (2023)
*Larp7*	cKO in GCs	Infertile	Blocked maturation of round spermatids	Mediate U6 snRNA 2′-O-methylation to ensure splicing fidelity	Wang *et al.* (2020c)
*Ldhc*	KO mice	Infertile	Reduced motility of sperm	May play a role in sperm motility	Odet *et al.* (2013)
*Lrrc46*	KO mice	Infertile	Short, coiled, and irregular flagella of sperm	Plays an important role in sperm flagellum biogenesis	Yin *et al.* (2022)
*Mael*	Mutant mice	Infertile	Malformed acrosome and flagellum of sperm	Participate in pachytene piRNA biogenesis and the translation of spermiogenic mRNAs	Castaneda *et al.* (2014)
*Mkrn2*	KO mice	Infertile	Low sperm count, poor motility and abnormal head morphology	Regulate the expression levels of *Odf2*	Qian *et al.* (2016)
*Msy4*	Transgenic mice	Infertile	Severe defects in sperm morphogenesis	Regulate the translational repression of the protamine 2 mRNAs	Giorgini *et al.* (2002)
*Odf2*	Mutant mice	Infertile	Abnormal spermatozoa with tailless heads and headless tails due to head-neck separation	Acts as a component of sperm tail outer dense fibers	Ito *et al.* (2019)
*Pdcl2*	KO mice	Infertile	Abnormal acrosome biogenesis, and reduced sperm motility	Regulate sperm acrosome formation	Fujihara *et al.* (2023)
*Piwil1 (Miwi)*	KO mice	Infertile	Arrested spermiogenesis	Regulate the stability of mRNAs of ACT (activator of CREM in testis) and CREM	Deng and Lin (2002)
*Rbm5*	Mutant mice	Infertile	Disrupted flagellar development, sperm head shaping and histone-to-protamine transitions	Regulate AS of pre-mRNAs in haploid male GCs	O’Bryan *et al.* (2013)
*Ranbp1*	KO mice	Infertile	Impaired spermatid elongation	Function as positive regulators for RanGAP activity	Nagai *et al.* (2011)
*Rnf17*	KO mice	Infertile	Arrested round spermatid differentiation	Participate in the repression of Ping-pong activity in pachytene piRNA biogenesis	Xiong *et al.* (2023)
*Setd2*	cKO in GCs	Infertile	Aberrant spermiogenesis with acrosomal malformation	Regulate H3K36me3 modification and the expression of ACRBP1 and protamines	Zuo *et al.* (2018)
*Sox30*	KO mice	Infertile	Arrested spermiogenesis	Regulate the expression of spermatid-specific protein-coding and long non-coding RNA genes	Zhang *et al.* (2018)
*Smg6*	cKO in GCs	Infertile	Immature acrosomal granule and acrosome fragmentation	Regulate nonsense-mediated RNA decay target mRNAs	Lehtiniemi *et al.* (2022)
*Smcp*	KO mice	Infertile	Reduced spermatids motility	Closely associate with the keratinous capsules of sperm mitochondria	Cannarella *et al.* (2025)
*Spata6*	KO mice	Infertile	Acephalic Spermatozoa	Regulate normal assembly of the sperm connecting piece and tight head-tail conjunction	Yuan *et al.* (2015)
*Spaca1*	KO mice	Infertile	Abnormally shaped sperm heads reminiscent of globozoospermia	Play a role in acrosome expansion and establishment of normal sperm morphology	Fujihara *et al.* (2012)
*Spink2*	KO mice	Infertile	Azoospermia	Encode a serine protease inhibitor to target acrosin	Kherraf *et al.* (2017)
*Tarbp2*	KO mice	Infertile	D severe oligospermia and spermatozoa with morphological abnormalities	Regulate proper translational activation of the mRNAs encoding the protamines	Theotoki *et al.* (2024)
*Tbpl1(Tlf)*	KO mice	Infertile	Complete arrest of spermiogenesis	Play a role at the transition from post-meiotic spermatids into spermatozoa	Martianov *et al.* (2001)
*Tsga10*	KO mice	Infertile	Reduced sperm motility	Involved in the correct arrangement of mitochondrial sheath	Luo *et al.* (2021)
*Tsga8*	KO mice	Infertile	Abnormal chromosomal distribution in round spermatids	Regulate nuclear condensation and histone-to-protamine transition	Kobayashi *et al.* (2021)
*Tsks*	KO mice	Infertile	Abnormal shape of sperm in the center and/or head	Regulate cytoplasmic elimination during spermiation	Shimada *et al.* (2023)
*Tdrd6*	KO mice	Infertile	Distorted chromatoid body architecture in round spermatids	Support long 3’-UTR triggered nonsense mediated mRNA decay and regulate miRNA expression.	Gao *et al.* (2024)
*Tdrd7*	KO mice	Infertile	Arrested spermiogenesis and defective acrosome development	Regulate dynamic ribonucleoproteins remodeling of chromatoid bodies	Tanaka *et al.* (2011)
	Mutant mice	Infertile	Arrested spermiogenesis	Participate in the posttranscriptional control of mRNAs	Lachke *et al.* (2011)
*Tulp2*	KO mice	Infertile	Defective sperm tail structures and reduced sperm motility	Regulate specific transcripts related to the cytoskeleton, apoptosis, RNA metabolism and biosynthesis, and energy metabolism	Zheng *et al.* (2021)
*Tssa*	KO mice	Infertile	Arrested spermiogenesis at step 6	Regulate the stabilization of *Miwi*-mediated mRNA	Chen *et al.* (2023)
*Ttc21a*	Mutant mice	Infertile	Defective flagella and the connecting piece of sperm	Regulate the sperm flagellar formation and intra-flagellar transport	Liu *et al.* (2019a)
*Wdr62*	cKO in GCs	Infertile	Misshapen spermatid heads with elongated manchettes	Involved in manchette removal	Ho *et al.* (2021)
*Ybx2 (Msy2)*	KO mice	Infertile	Misshapen and multinucleated condensed spermatids, and blocked spermatid elongation	Regulate transcription activation, stabilize and store mRNAs	Yang *et al.* (2005)
*Ythdf2*	cKO in GCs	Infertile	Malformation and impaired motility of spermatids	Mediate mRNA degradation and cellular differentiation	Lasman *et al.* (2020), Qi *et al.* (2022)
*Zrsr1*	Mutant mice	Infertile	Severe spermatogenic defects and abnormal spermatid morphology	Play a role in RNA splicing	Horiuchi *et al.* (2018)
*Zmynd15*	KO mice	Infertile	Depleted elongating and elongated spermatids	Act as a transcriptional repressor through interaction with histone deacetylases	Yan *et al.* (2010)
*Zpbp (Zpbp1)*	KO mice	Infertile	Abnormal round-headed sperm morphology and no forward sperm motility	Play a role in acrosome biogenesis and sperm morphogenesis	Lin *et al.* (2007)

AS, alternative splicing; BTB, blood-testis barrier; cKO, conditional knockout; DSB, DNA double-strand break; GCs, germ cells; KO, knockout; MUT, mutant; piRNA, Piwi-interacting RNA; Pro-SG, progenitor spermatogonia; SCs, Sertoli cells; SSCs, spermatogonial stem cells.

### Roles of RBPs in mouse spermatocytogenesis

Spermatocytogenesis, the first stage of spermatogenesis, requires a tightly orchestrated balance between self-renewal, proliferation, and differentiation of spermatogonial stem cells (SSCs) and their progeny. This progression depends on RBPs that regulate RNA stability, alternative splicing, and chromatin remodeling to maintain the transcriptomic and epigenetic landscape of germ cells.

#### Self-renewal and maintenance of SSCs

The maintenance of SSCs is essential for the continuous production of male germ cells. RBPs such as NANOS2 (Sada *et al.*, 2009), NANOS3 (Tsuda *et al.*, 2003), DAZL (Li *et al.*, 2019a), and DDX4 (Kuramochi-Miyagawa *et al.*, 2010) play pivotal roles in maintaining the balance between self-renewal and differentiation. NANOS2, expressed in undifferentiated SSCs, prevents premature differentiation by suppressing differentiation-promoting pathways. Mice lacking NANOS2 exhibit gradual depletion of SSCs, ultimately leading to germ cell loss. In contrast, NANOS3 is required for the survival of early spermatogonia and cooperates with DND1 to suppress the entry of germ cell lineage (Wang *et al.*, 2025). Its knockout results in a complete loss of germ cells, emphasizing its indispensable role in sustaining the germline. DAZL is a master translational regulator during spermatogenesis. The postnatal stage-specific deletion of *Dazl* caused complete male sterility with gradual loss of SSCs, meiotic arrest, and spermatid arrest in mice (Li *et al.*, 2019a). DDX4 plays crucial role in the early phase of the ping-pong amplification cycle. Loss of DDX4 results in defective expression of piRNA and male infertility in mice (Kuramochi-Miyagawa *et al.*, 2010).

PRMT1 (Azhar *et al.*, 2023), DDX20 (Zou *et al.*, 2024), and HNRNPU regulate SSC establishment and the transition to progenitor spermatogonia (Pro-SG). PRMT1 modulates the arginine methylation of splicing factors, ensuring proper transcript processing and stability. Its absence leads to impaired SSC differentiation and maintenance. Similarly, DDX20 governs the cell cycle reentry of Pro-SG and the establishment of the SSC pool. Knockout models lacking DDX20 demonstrate a complete failure to form Pro-SG or SSCs, underscoring its essential role in the earliest stages of spermatocytogenesis. HNRNPU stabilizes transcripts and remodel chromatin to ensure proper establishment of the SSC pool and the transition from T1-ProSG to T2-ProSG (Wen *et al.*, 2021, 2024b). Its loss leads to the accumulation of undifferentiated Pro-SG and failed differentiation.

Alternative splicing, a critical process for generating transcriptome diversity, is tightly regulated by RBPs such as SRSF1 and SRSF10 (Liu *et al.*, 2022a; Sun *et al.*, 2024). SRSF1 knockout mice exhibit the complete absence of germ cells due to defective homing and survival of precursor SSCs. SRSF10, essential for the expansion of Pro-SG, regulates splicing programs critical for their proliferation. Loss of SRSF10 disrupts spermatogonial differentiation and leads to reduced germ cell numbers.

#### Proliferation and differentiation of spermatogonia

The proliferation of spermatogonia and their differentiation into primary spermatocytes require the coordinated regulation of RNA metabolism and chromatin remodeling. RBPs such as DIS3L2 and UHRF1 safeguard the transcriptome and epigenome during this stage. DIS3L2 degrades aberrant RNAs and suppresses retrotransposon activity to maintain genomic integrity. Loss of DIS3L2 results in impaired differentiation and increased apoptosis of spermatogonia (Li *et al.*, 2024b). Similarly, UHRF1, a multifunctional RBP, maintains SSC homeostasis with epigenetic regulation. Knockout models lacking UHRF1 exhibit SCOS by disrupting the interaction with snRNA in spermatogonia (Zhou *et al.*, 2022).

DDX5 (Legrand *et al.*, 2019), DHX36 (Gao *et al.*, 2015), and CWF19L2 regulate key gene expression in spermatogonial differentiation. Deficiency of DDX5 leads to rapid and profound depletion of spermatogonia, while knockout of DHX36 reduces spermatogonial differentiation efficiency by downregulating c-KIT expression. CWF19L2 ensures the correct splicing of key transcripts during early spermatogenesis, with its knockout resulting in increased apoptosis and reduced differentiation (Wang *et al.*, 2024a).

Additionally, METTL16 and RBM46 play central roles in proper spermatogonial differentiation and meiosis initiation. METTL16 controls the mitosis-to-meiosis fate decision through m^6^A modifications, and its disruption results in a failure of RNA modifications in spermatogonial differentiation (Ma *et al.*, 2024). RBM46 regulates the stability and translation of key differentiation transcripts. Knockout mice lacking RBM46 exhibit spermatogonial arrest and SCOS, underscoring its essential role in the completion of spermatogonial differentiation (Peart *et al.*, 2022).

The roles of RBPs in spermatocytogenesis are diverse yet interconnected, spanning SSC maintenance, spermatogonial proliferation, and differentiation. Their functional roles in RNA stability, alternative splicing, and chromatin remodeling ensure the proper transcriptional and post-transcriptional regulation during early germ cell development. RBP genes, such as NANOS2, NANOS3, DAZL, DDX4, PRMT1, DDX20, HNRNPU, SRSF1, and SRSF10, regulate self-renewal and maintenance of SSCs. In contrast, certain RBP genes, DIS3L2, UHRF1, DDX5, DHX36, CWF19L2, METTL16, and RBM46, are essential for proliferation and differentiation of spermatogonia, underscoring their potential as molecular markers and therapeutic targets in male fertility regulation.

### Role of RBPs in mouse spermatidogenesis

Spermatidogenesis encompasses the entire process by which primary spermatocytes undergo two sequential meiotic divisions to form haploid round spermatids. This process involves homologous chromosome pairing, DNA double-strand breaks (DSBs), synaptonemal complex formation, DSB repair, crossover formation, homologous recombination, and meiotic sex chromosome inactivation (MSCI) (Hinch *et al.*, 2023). This stage is tightly orchestrated by RBPs through RNA modification and piRNA biogenesis. Insights from gene knockout models in mice provide a detailed understanding of the indispensability of RBPs in spermatidogenesis.

#### Homologous pairing, DSBs, and synaptonemal complex formation

During the prophase of meiosis I, the homologous chromosome pairing, DSBs, and synaptonemal complex formation are dependent on RBPs, such as SYCP2, TEX11, SYCE1, NAT10, TDP-43 (*Tardbp*), RBM46, YTHDC2, MEIOC, PRRC2A, TDRKH (TDRD2), FKBP6, GTSF1.

SYCP2 is a major component of the axial/lateral elements in synaptonemal complexes during meiotic prophase, promoting the assembly of synaptonemal complexes. Its deficiency results in chromosomal synapsis disruption and meiotic arrest (Yang *et al.*, 2006). TEX11 promotes initiation and/or maintenance of synapsis through interacting with SYCP2. Loss of TEX11 function causes chromosomal asynapsis, leading to elimination of spermatocytes (Yang *et al.*, 2008). SYCE1 is a major component of the transverse central element of synaptonemal complex. Null mutation in mouse Syce1 disrupts synapsis during meiotic prophase (Bolcun-Filas *et al.*, 2009). NAT10 participates in post-transcriptional RNA modifications. Germ cell-specific ablation of *Nat10* or *Tardbp* severely inhibits meiotic entry and leads to defects in homologous chromosome synapsis (Campbell *et al.*, 2021; Chen *et al.*, 2022).

RBM46/YTHDC2/MEIOC complex, a major post-transcriptional regulator, is essential for a successful meiotic program in the mammalian germline. Despite *Rbm46* knockout mice exhibit abnormal spermatogonial differentiation, germ cell-specific *Rbm46* knockout mice using neurog-cre show aberrant DSBs and synaptonemal complex formation (Qian *et al.*, 2022). MEIOC maintains the extended meiotic prophase I in mice. The deficiency of MEIOC causes failed transition to meiotic cell cycle program (Soh *et al.*, 2017). YTHDC2, a m6A reader, modulates the levels of m6A-modified germline transcripts. The germ cells of *Ythdc2* knockout mice arrest at the zygotene stage (Hsu *et al.*, 2017; Wojtas *et al.*, 2017). PRRC2A, also a m6A reader, regulates mRNA translation in meiotic cell cycle. *Prrc2a*-deficient mice exhibit XY asynapsis and impaired meiotic sex chromosome inactivation in late-prophase spermatocytes (Tan *et al.*, 2023b).

TDRKH specifically recruits MIWI, but not MILI, to engage the piRNA pathway (Ding *et al.*, 2019). *Tdrkh* mutants display defects in forming synaptonemal complexes at the zygotene stage (Saxe *et al.*, 2013). FKBP6 mediates piRNA metabolic process and transposable elements repression. Loss of *Fkbp6* results in abnormal pairing and misalignments between homologous chromosomes (Crackower *et al.*, 2003). GTSF1 regulates the suppression of retrotransposon transcription. The loss of *Gtsf1* leads to the accumulation of DNA damage and meiotic arrest before the zygotene stage (Yoshimura *et al.*, 2009).

#### DSB repair, crossover formation, homologous recombination, and MSCI

The synaptonemal complex serves as a structural scaffold that facilitates DSB repair and crossover formation. RBPs such as BRDT1, CNOT4, GGNBP2, TCFL5, HSF5, HFM1, MDC1, UHRF1, TDRD1, TDRD9, MOV10L1 orchestrate these processes.

BRDT is an epigenetic regulator for chromatin organization, crossover formation, and MSCI. Loss of BRDT blocks the progression of spermatocytes into meiosis I, resulting in a complete absence of post-meiotic cells (Manterola *et al.*, 2018). CNOT4 integrates mRNA degradation with DNA damage repair processes, facilitating XY chromosome pairing and crossover. Loss of CNOT4 disrupts homologous recombination, impairing spermatogenic progression (Dai *et al.*, 2021). GGNBP2, in collaboration with GGN1, supports crossover formation. Knockout of GGNBP2 results in reduced crossover frequency and meiotic arrest, underscoring its role in ensuring recombination efficiency (Guo *et al.*, 2018). TCFL5 mediates the pachytene to diplotene transition during spermatogenesis. TCFL5 deficiency causes the condensation of the sexual body and abnormal separation of synaptonemal complex (Galan-Martinez *et al.*, 2022).

HSF5 modulates the transcriptome to ensure meiotic progression, including key genes *Sycp2*, *Hfm1*, and *Mdc1*. *Hsf5* knockout mice exhibit defects in crossover formation, sex chromosome synapsis, and MSCI (Liu *et al.*, 2024a; Luo *et al.*, 2024). HFM1, a DNA helicase, is required for crossover formation. *Hfm1* knockout in mice leads to aberrant recombination (Guiraldelli *et al.*, 2013). MDC1 directs chromosome-wide silencing of the sex chromosomes in male germ cells. *Mdc1*-null mice shows meiotic arrest (Ichijima *et al.*, 2011) ZFP541-KCTD19 complex is essential for pachytene progression. Depletion of *Zfp541* or *Kctd19* in mice causes abnormal DSB repair and asynapsis of the XY chromosomes (Li *et al.*, 2022b; Xu *et al.*, 2022b).

UHRF1 is responsible for DSB repair by regulating DNA methylation and retrotransposon silencing. UHRF1 deficiency leads to meiosis arrest and male infertility (Dong *et al.*, 2019; Pan *et al.*, 2020). Similarly, TDRD1 and TDRD9 also regulate retrotransposon silencing. TDRD1 interacts with MILI, while TDRD9 forms a complex with MIWI2 (Shoji *et al.*, 2009; Wang *et al.*, 2009). *Tdrd1* knockout mice exhibit meiotic arrest at leptotene or zygotene stage through disrupting intermitochondrial cement assembly and piRNA biogenesis (Gao *et al.*, 2024). TDRD9 depletion results in defective piRNA maturation, transposon derepression, and ultimately, spermatogenic failure (Wenda *et al.*, 2017). *Mov10l1* knockout mice show activation of LTR and LINE-1 retrotransposons, followed by cell death, causing male infertility and a complete block of spermatogenesis at early prophase of meiosis I (Frost *et al.*, 2010).

### Roles of RBPs in mouse spermiogenesis

Spermiogenesis, the final stage of spermatogenesis, transforms haploid round spermatids into mature spermatozoa through a series of tightly coordinated molecular and morphological changes. These transformations include chromatin remodeling, HTP transition, acrosome biogenesis, and flagellar assembly. These changes equip sperm with motility and fertilization potential, driven by precise post-transcriptional regulation of RBPs. Insights from knockout mouse models reveal the indispensable roles of RBPs in spermiogenesis, highlighting their multifaceted regulatory mechanisms and associated infertility phenotypes.

#### Chromatin remodeling and HTP transition

Post-meiotic chromatin remodeling and the HTP transition represent essential processes in spermiogenesis, culminating in the formation of a highly compacted and transcriptionally inert chromatin structure. This transition involves the orderly replacement of histones by transition proteins (TNPs) and subsequently by protamines (PRMs), and is tightly regulated by multiple RBPs that orchestrate both transcriptional and post-transcriptional events.

Among these, CCER1 has emerged as a key regulator of chromatin remodeling and the HTP transition. Functioning as a nuclear phase-separated condensate, CCER1 enhances the transcription of *Tnp1/2* and *Prm1/2*, and facilitates multiple histone epigenetic modifications required for chromatin reorganization. Loss of CCER1 results in defective chromatin condensation and male infertility (Qin *et al.*, 2023). In parallel, DDX43 modulates chromatin structure through dynamic RNA regulatory mechanisms. Mice lacking *Ddx43* exhibit impaired HTP transition and abnormal chromatin compaction (Tan *et al.*, 2023a). Similarly, TSG8 is critical for mediating nuclear condensation through regulation of TNP1 and PRM1; its deletion causes defective nuclear shaping and aberrant sperm head morphology (Kobayashi *et al.*, 2021).

In addition to these regulators, EWSR1 contributes to chromatin remodeling by controlling the synthesis of spermiogenesis-related mRNAs. Knockout of *Ewsr1* leads to disrupted chromocenter formation, abnormal DNA condensation, and failure of spermatid elongation at the round spermatid stage (Tian and Petkov, 2021). RBM5 regulates alternative splicing in haploid germ cells. Loss of *Rbm5* results in impaired HTP transition and disrupted head shaping (O’Bryan *et al.*, 2013). TARBP2 is required for the translational activation of protamine-encoding mRNAs; its absence delays histone displacement, further impeding chromatin compaction (Zhong *et al.*, 1999). PIWIL1 (MIWI) maintains the stability of spermiogenic transcripts. *Miwi*-null mice exhibit spermatogenic arrest at the round spermatid stage, accompanied by a complete failure in nuclear condensation (Deng and Lin, 2002). Finally, MSY4 acts as a translational repressor of stored *Prm2* mRNAs. Dysregulated expression of *Msy4* disturbs the precise timing of PRM2 translation, resulting in severe morphological abnormalities in spermatozoa (Giorgini *et al.*, 2002).

#### Acrosome biogenesis

Acrosome biogenesis is an RNP-regulated vesicular trafficking process that involves the coordinated fusion of proacrosomal vesicles and the assembly of a specialized membrane-bound organelle essential for sperm function (He *et al.*, 2025). This process is tightly regulated by RBPs, which govern transcriptome, piRNA pathway, and nonsense-mediated mRNA decay (NMDs).

ADAD2 forms a repressive complex with RNF17 in P-bodies to modulate pachytene piRNA and acrosome formation. Knockout of *Adad2* or *Rnf17* results in arrested round spermatid differentiation and abnormal acrosome morphology (Xiong *et al.*, 2023). ACTL7A modulates acrosome-acroplaxome-manchette formation and autophagy inhibition via PI3K/AKT/mTOR signaling pathway. Loss of *Actl7a* leads to defective acrosome architecture and impaired sperm head shaping (Ferrer *et al.*, 2023; Zhang *et al.*, 2023). SPNR (*Strbp*) localizes to the manchette in developing spermatids. Mice deficient for SPNR show sperm morphological abnormalities (Pires-daSilva *et al.*, 2001).

CCDC136 (Geng *et al.*, 2016), PDCL2 (Fujihara *et al.*, 2023), and ZPBP1 (Lin *et al.*, 2007) are essential for acrosome biogenesis. Knockout mouse models of *Ccdc136*, *Pdcl2*, and *Zpbp1* exhibit infertility due to severe acrosomal malformations. Mechanistically, CCDC136 regulates the expression of SPACA1, a critical RBP involved in acrosome expansion and nuclear-acrosomal junction formation. Disruption of *Spaca1* causes disorganized acrosomal structure and abnormal sperm head morphology (Fujihara *et al.*, 2012).

CYLC2, a calyx component, is critical for anchoring the acrosome to the nuclear envelope. *Cylc2*-deficient mice exhibit acrosomal detachment, distorted nuclear shaping, and defective sperm head morphology (Schneider *et al.*, 2023). SETD2, a histone methyltransferase, regulates H3K36me3 modification in spermiogenesis. Knockout *Setd2* in germ cells causes acrosomal malformation through decreasing mRNA expression of *Acrbp1* and protamines (Zuo *et al.*, 2018). Similarly, CCIN contributes to the structural organization of the sperm calyx. Disruption of *Ccin* causes several sperm abnormalities, including defective acrosomes and misassembled tails (Fan *et al.*, 2022).

SMG6, a RNP component involved in NMDs, cooperates with PIWIL1 to modulate many genes in round spermatids. *Smg6*-deficient mice exhibit severe acrosomal defects by regulating germline transcriptome in chromatid bodies (CBs) (Lehtiniemi *et al.*, 2022). TDRD6 and TDRD7 regulate the dynamic RNP remodeling of CBs. The absence of TDRD6 or TDRD7 results in distorted CBs and acrosomal developmental failure (Lachke *et al.*, 2011; Fanourgakis *et al.*, 2016).

SOX30 controls the expression of spermatid-specific protein-coding and long non-coding RNA genes. In *Sox30*-null mice, spermatogenesis is arrested at step 3 of round spermatid development, and the fusion of proacrosomal vesicles fails to generate a single acrosomal organelle (Zhang *et al.*, 2018). MAEL, a piRNA pathway component, is required for translation of spermiogenic mRNAs, encoding both acrosomal and flagellar proteins. *Mael*-deficient mice show spermiogenic arrest with combined acrosomal and flagellar malformations (Castaneda *et al.*, 2014). BOULE may also regulate the translation of key regulators during spermiogenesis. *Boule*-deficient mice show arrested round spermatid differentiation (VanGompel and Xu, 2010).

#### Flagellum assembly

The sperm flagellum is a highly specialized structure responsible for motility and fertilization competence. Its formation requires the coordinated assembly of axoneme, outer dense fibers, the fibrous sheath, and mitochondrial sheath. A variety of RBPs orchestrate these processes.

CEP131 and TTC21A are involved in the regulation of intraflagellar and intramanchette trafficking, which are critical for axoneme extension. *Cep131*-mutant mice exhibit defective flagellar morphology (Hall *et al.*, 2013). Deletion of TTC21A causes structural defects in both the flagellum and connecting piece of spermatozoa (Liu *et al.*, 2019a). Similarly, LRRC46, which accumulates at the midpiece of the flagellum, is indispensable for flagellum assembly. *Lrrc46*-deficient mice are infertile due to flagellar deformities (Yin *et al.*, 2022).

Axonemal dynein arms are key motor complexes for flagellum assembly. DNAH2, a component of the inner dynein arm, is crucial for flagellar axoneme. *Dnah2*-deficient mice exhibit multiple MMAF phenotype (Hwang *et al.*, 2021). Likewise, Loss of DNAH3 results in asthenoteratozoospermia (Meng *et al.*, 2024). DNAH8 is a force-generating element of the outer dynein arms. *Dnah8*-null mice exhibit disrupted axonemal assembly (Liu *et al.*, 2020).

MKRN2 modulates the expression of *Odf2*, a structural component of the outer dense fibers. Mice lacking *Mkrn2* produce sperm with disorganized outer dense fibers, accompanied by head and acrosome abnormalities (Qian *et al.*, 2016). AKAP3 and AKAP4, major constituents of the fibrous sheath, play indispensable roles in the structural integrity and functional maturation of the sperm flagellum. *Akap3* deficiency leads to mislocalization of sperm proteins, including aberrant accumulation of RNA metabolism and translation factors (Xu *et al.*, 2020). *Akap4*-null mice display impaired fibrous sheath assembly and abnormal sperm morphology (Fang *et al.*, 2019).

The connecting piece, which anchors the flagellum to the sperm head, is also critical for flagellar function. SPATA6 is required for the assembly of this region and for proper head–tail conjunction. *Spata6*-deficient males are sterile due to formation of acephalic spermatozoa (Yuan *et al.*, 2015). TSGA10 is essential for mitochondrial sheath assembly—a key process supporting energy production for motility. *Tsga10*-deficient mice are infertile and show disrupted mitochondrial sheath organization, leading to reduced ATP production and compromised sperm motility (Luo *et al.*, 2021). SLIRP modulates mitochondrial transcription. Knockout models reveal defective mitochondrial morphology and reduced motility (Colley *et al.*, 2013). TULP2, regulates transcripts involved in cytoskeletal organization, apoptosis, and energy metabolism. Loss of *Tulp2* impairs ATP production and results in defective tail structures (Zheng *et al.*, 2021).

In addition to transcriptional regulators, post-transcriptional regulators also contribute to sperm flagellum assembly. ZRSR1, a splicing factor, contributes to post-transcriptional regulation during spermiogenesis. *Zrsr1*-deficient mice display multiple sperm morphological defects, including abnormal head assembly, and structural abnormalities in the neck, midpiece, and tail regions (Horiuchi *et al.*, 2018).

Cytoplasmic elimination is also a crucial part during spermiogenesis. TSKS, a component of nuage in spermatids, plays an essential role in regulating this process. Knockout of *Tsks* leads to failed cytoplasmic elimination during spermiogenesis, resulting in spermatozoa with residual cytoplasm and abnormal head morphology (Shimada *et al.*, 2023).

Taken together, RBPs play indispensable roles in the transcriptional and post-transcriptional regulation during spermiogenesis. From chromatin remodeling and HTP transition to acrosome biogenesis and flagellar assembly, RBPs ensure the precise temporal expression and localization of mRNAs. Disruption of these regulatory processes often leads to spermatogenic arrest and malformed spermatozoa, as evidenced by phenotypes observed in multiple RBP-deficient mouse models. A variety of RBPs—acting as splicing regulators (e.g. RBM5, ZRSR1), translational repressors (e.g. MSY4), and piRNA pathway components (e.g. PIWIL1, MAEL)—highlight the complexity and specialization of the regulatory networks guiding spermatid differentiation.

### Identification of 38 candidate RBP genes in male infertility

**Figure 5.**
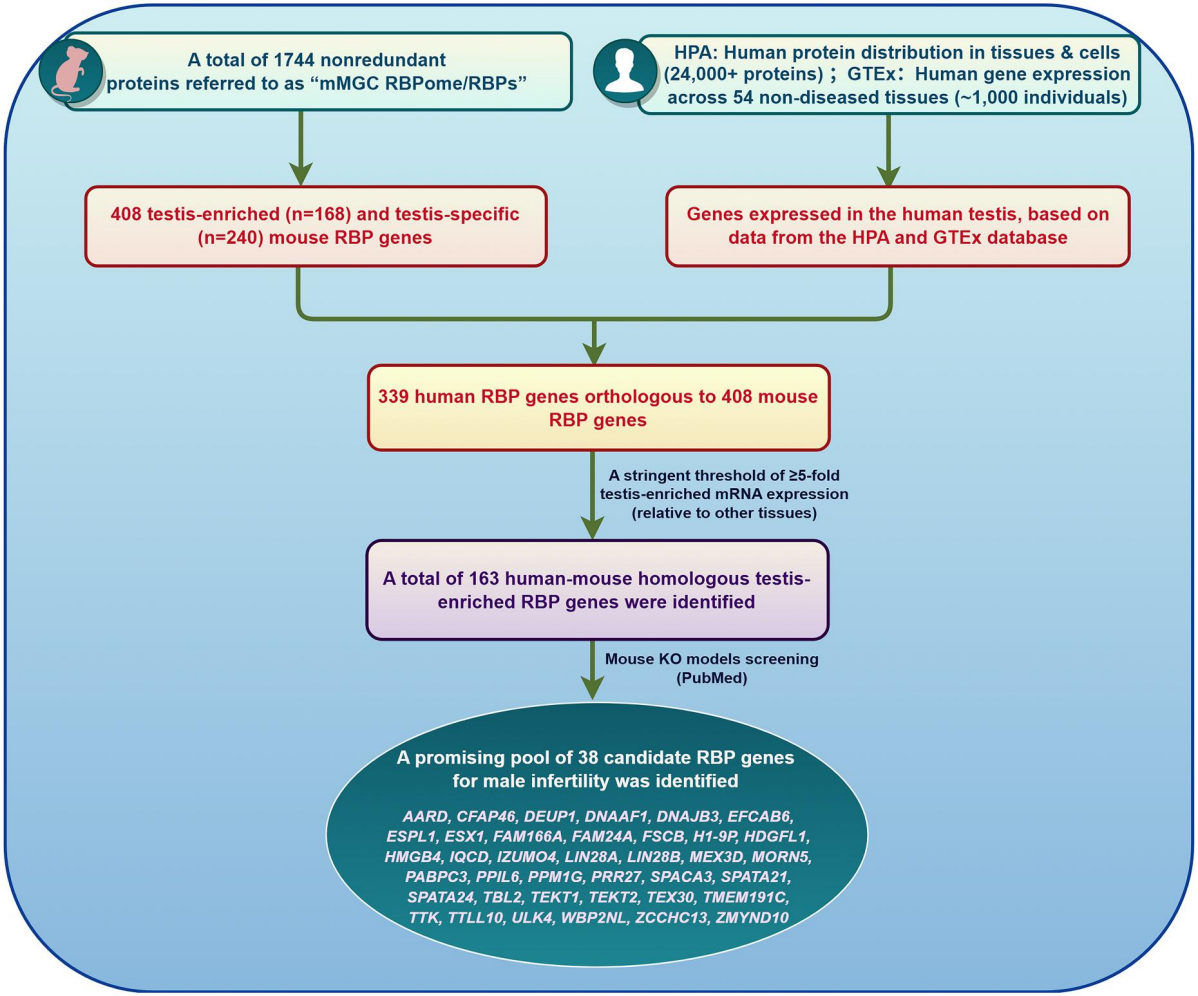
**Workflow for identifying 38 candidate RNA-binding protein (RBP) genes potentially implicated in male infertility.** This flowchart illustrates the stepwise strategy used to prioritize 38 candidate RBP genes for further investigation in male reproductive biology. The process began with mining the ‘male mouse germ cell RBPome’ database, comprising 408 RBPs enriched (n=168) and specific (n=240) to mouse testes. Human orthologs were identified through homology mapping and cross-referenced with expression data from the Genotype-Tissue Expression and Human Protein Atlas databases. A total of 339 RBPs were found to be expressed in human testicular tissue. Applying a selection criterion of testis-specific enrichment (≥5-fold higher mRNA expression in testis compared to other tissues), 163 testis-enriched human-mouse homologous RBP genes were retained. A comprehensive literature search (PubMed) was conducted to evaluate the functional relevance of these genes in male fertility. Of the 163 RBPs, 125 had previously been associated with reproductive phenotypes in mouse models. The remaining 38 genes (comprising 3 classical, 3 non-classical, and 32 novel RBPs) lacked knockout mouse models, representing a prioritized subset for future functional validation. GTEx, Genotype-Tissue Expression； HPA, Human Protein Atlas; KO, knockout; mMGC, male mouse germ cell.

To systematically uncover previously uncharacterized RBP genes potentially implicated in male infertility, we leveraged a comprehensive RBP atlas published in *Science*, which identified 1744 testicular RBPs in mice (Li *et al.*, 2024c). From this dataset, we screened 408 RBPs with testis-enriched expression profiles in mice. Cross-referencing with the GTEx project (GTEx Consortium, 2015) and the HPA (Uhlen *et al.*, 2015), we determined that 339 of these genes have human orthologs. Human testis-enriched RBPs were defined as those exhibiting at least 5-fold higher in the testis compared to all other tissues, resulting in a curated list of 163 RBPs with conserved testis-enriched expression across species. These included 17 classical RBPs, 26 non-classical RBPs, and 120 novel RBPs (Fig. 5).

**Figure 6.**
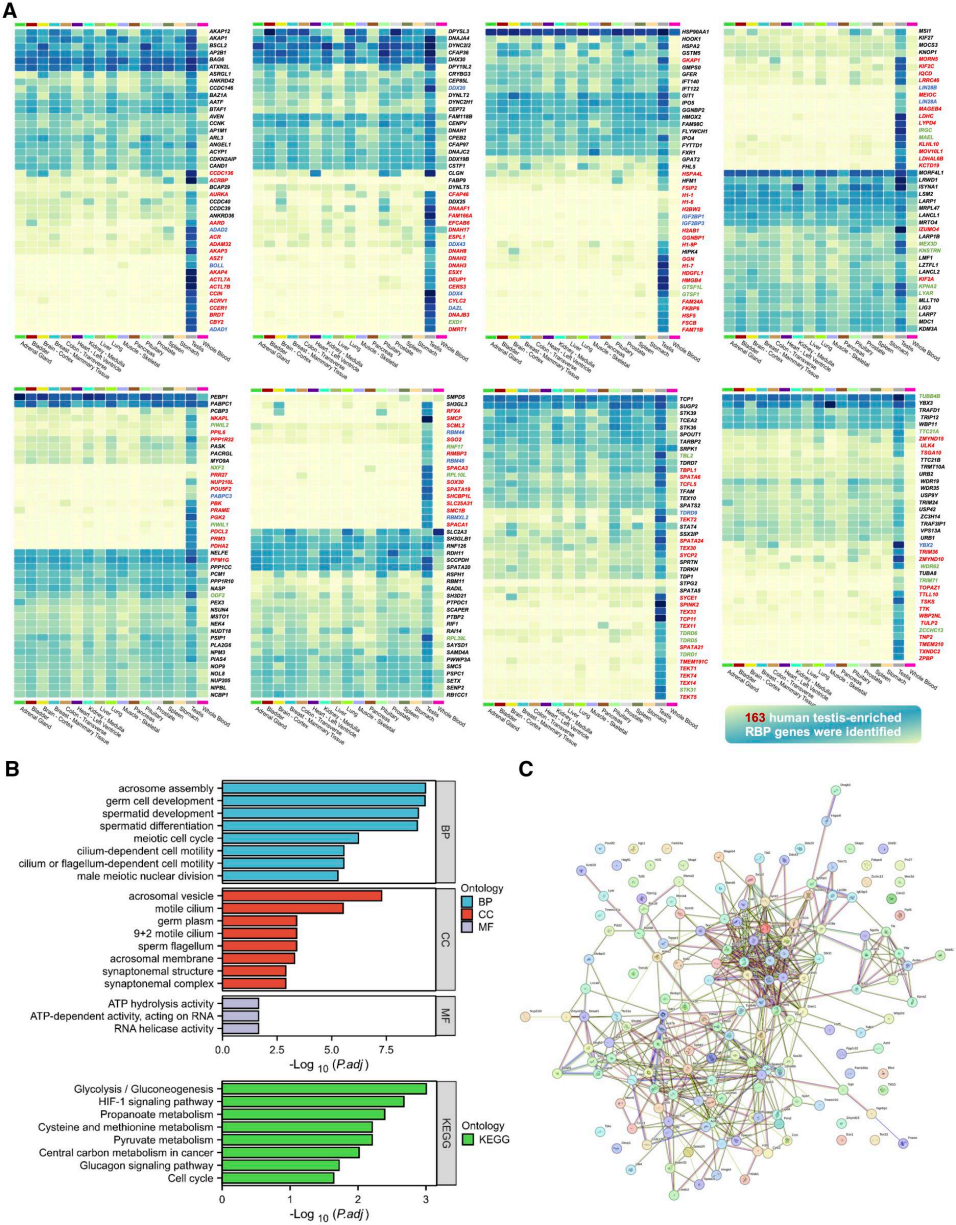
**Integrated analysis of 163 testis-enriched RNA-binding proteins (RBPs): gene expression, functional enrichment, and protein-protein interaction (PPI).** **(A) Heatmap of RBP Gene Expression across Human Tissues (GTEx).** Normalized expression levels of 163 testis-enriched RBP genes are shown across multiple human tissues using data from the GTEx database. Genes are arranged on the *y*-axis and tissues on the *x*-axis. Color intensity reflects expression levels (low: light yellow; high: dark blue). Genes with testis-specific enrichment (>5-fold higher expression in testes relative to other tissues) are highlighted by RBP classification: classical (blue, n=17), non-classical (green, n=26), and novel (red, n=120). This heatmap illustrates the tissue-specific expression landscape of RBPs, with a particular emphasis on testis-predominant expression. **(B) Gene Ontology (GO) and Kyoto Encyclopedia of Genes and Genomes (KEGG) Pathway Enrichment Analyses.** GO terms and KEGG pathways enriched among the 163 testis-enriched RBPs are depicted. The GO analysis includes biological process (BP), cellular component (CC), and molecular function (MF) categories, highlighting roles in spermatogenesis, RNA processing, and subcellular localization. KEGG analysis identifies key signaling and metabolic pathways relevant to testicular function and male fertility. **(C) PPI network.** A PPI network of testis-enriched RBPs was constructed using the Search Tool for the Retrieval of Interacting Genes/Proteins database. Nodes represent individual RBPs, and edges indicate predicted or known interactions, weighted by confidence scores. This network provides insight into the potential cooperative functions and regulatory hubs of RBPs involved in spermatogenesis and testicular physiology.

Tissue-specific heatmap analyses confirmed the strong testicular specificity of these RBPs, supporting their potential roles in spermatogenesis. GO enrichment analyses revealed that significant associations with RNA splicing, acrosome assembly, germ cell development, and ATP hydrolysis activity. KEGG pathway analysis further implicated these RBPs in key testicular biological pathways, including glycolysis/gluconeogenesis, HIF-1 signaling pathway, and propanoate metabolism. Moreover, a protein–protein interaction (PPI) network constructed via the STRING (https://string-db.org) database demonstrated that these RBPs are functionally interconnected within molecular networks essential for germ cell development and maturation (Fig. 6) (Szklarczyk *et al.*, 2023).

Next, we performed a comprehensive PubMed search to determine the availability of knockout mouse models for these 163 testis-enriched RBPs. Among these, 125 genes have reported male reproductive phenotypes, indicating established roles in spermatogenesis or fertility. In contrast, 38 RBPs—including 3 classical, 3 non-classical, and 32 novel RBPs—currently lack knockout model data, and their roles in male reproduction remain uncharacterized. To gain initial insight into the potential functions of these 38 candidate RBP genes, we analyzed their mRNA and protein expression patterns in human testes using data from GTEx and HPA datasets. Additionally, we retrieved their functional annotations from Universal Protein (UniProt) database and assessed their evolutionary conservation between human and mouse (Table 4), providing a foundational resource for future functional studies (UniProt, 2023).

**Table 4.** Identification of 38 uncharacterized candidate RNA-binding protein genes in male infertility.

Gene	mRNA level in human testis (GTEx)	Protein expression in human testis (HPA)	Mouse vs human conservation (Uniprot)	Functions	Ref.
*AARD*	45.83	N.R.	58.17%	Interacts with AR and is directly regulated by AR in mouse Sertoli cells	Geng *et al.* (2017)
*CFAP46*	32.59	N.R.	47.59%	Plays a role in cilium movement as part of the central apparatus of the cilium axoneme	Wohlgemuth *et al.* (2024)
*DEUP1*	23.78	N.R.	80.70%	Enables massive *de novo* centriole biogenesis for vertebrate multiciliogenesis	Zhao *et al.* (2013)
*DNAAF1*	79.79	Positive	66.50%	Plays a role in assembly of distinct dynein-arm complexes	Loges *et al.* (2009)
*DNAJB3*	63.17	N.R.	69.44%	Associates with the acrosome and centrosome in mouse germ cells	Berruti and Martegani (2002)
*EFCAB6*	49.03	N.R.	66.24%	Interacts with CFAP52 and plays a role in the stability of sperm DMTs	Tai *et al.* (2023)
*ESPL1*	19.03	Negative	77.40%	Plays a central role in the chromosome segregation	Hauf *et al.* (2001)
*ESX1*	19.87	Positive	35.43%	Acts as a potential candidate responsible for male infertility in nonobstructive azoospermia	Malcher *et al.* (2023)
*FAM166A*	138.00	Positive	78.66%	Acts as a part of the dynein-decorated DMTs in flagellum axoneme	Zhou *et al.* (2023)
*FAM24A*	36.68	Positive	50.00%	Unknown	Unknown
*FSCB*	71.04	Positive	45.52%	Unknown	Unknown
*H1-9P*	29.51	N.R.	41.45%	Participates in chromatin remodeling during mammalian spermiogenesis	Yan *et al.* (2003)
*HDGFL1*	232.40	Positive	48.55%	Unknown	Unknown
*HMGB4*	517.90	Positive	72.93%	Plays a major role in sensitizing testicular germ cell tumors to cisplatin, consistent with shielding of platinum-DNA adducts from excision repair	Catena *et al.* (2009)
*IQCD*	79.58	Positive	67.94%	Acts as a new acrosomal protein involved in the acrosome reaction and fertilization	Zhang *et al.* (2019)
*IZUMO4*	753.30	Positive	75.69%	Unknown	Unknown
*LIN28A*	12.65	Positive	96.65%	Binds to meiotic gene transcripts and modulates their translation in male germ cells	Wang *et al.* (2020b)
*LIN28B*	4.75	Negative	86.64%	Suppresses the miRNA biogenesis, including that of let-7 and possibly of miR107, miR-143 and miR-200c	Piskounova *et al.* (2011)
*MEX3D*	151.80	Positive	79.90%	Promotes cervical carcinoma tumorigenesis by destabilizing TSC22D1 mRNA	Zheng *et al.* (2022)
*MORN5*	138.00	N.R.	78.88%	Interacts with the BMP and TGFβ pathways during craniofacial development	Cela *et al.* (2016)
*PABPC3*	7.99	Positive	77.96%	Participates in the modulation of GNRH1 mRNA stability and translation in mammalian puberty	Li *et al.* (2021)
*PPIL6*	19.45	Positive	71.70%	Probable inactive PPIase with no peptidyl-prolyl cis-trans isomerase activity	Davis *et al.* (2010)
*PPM1G*	639.50	Positive	93.91%	Regulates protein translation and cell growth by dephosphorylating 4E binding protein 1	Liu *et al.* (2013)
*PRR27*	1.68	Negative	38.53%	Unknown	Unknown
*SPACA3*	186.10	Positive	62.98%	Participates in sperm-egg plasma membrane adhesion and fusion during fertilization	Herrero *et al.* (2005)
*SPATA21*	12.59	N.R.	66.02%	Unknown	Unknown
*SPATA24*	164.20	Positive	90.73%	Contributes to the precise timing of the molecular events in male germ cells, specifically by linking transcription to chromatin remodeling in round spermatids	Brancorsini *et al.* (2008)
*TBL2*	173.60	N.R.	88.01%	Unknown	Unknown
*TEKT1*	33.40	Positive	82.54%	Forms filamentous polymers in the walls of ciliary and flagellar microtubules	Gui *et al.* (2022)
*TEKT2*	142.00	Positive	83.72%	Associates with the pathogenesis of the testicular germ cell tumors	Liu *et al.* (2023d)
*TEX30*	82.96	Positive	96.00%	Unknown	Unknown
*TMEM191C*	37.81	N.R.	65.57%	Unknown	Unknown
*TTK*	28.85	Positive	75.69%	Participates in mitotic spindle assembly checkpoint signaling and in the repair of incorrect mitotic kinetochore-spindle microtubule attachment	Jelluma *et al.* (2008), Maciejowski *et al.* (2017)
*TTLL10*	82.00	N.R.	65.80%	Function as polyglycylase which modifies both tubulin and non-tubulin proteins	Ikegami and Setou (2009)
*ULK4*	29.85	Positive	82.59%	May be involved in the remodeling of cytoskeletal components, such as alpha-tubulin	Lang *et al.* (2014)
*WBP2NL*	31.80	Positive	66.45%	Unknown	Unknown
*ZCCHC13*	49.60	Positive	59.51%	Positively regulates the AKT/MAPK/c-MYC pathway and likely functions to promote sperm proliferation	Li *et al.* (2018)
*ZMYND10*	609.00	N.R.	97.00%	Plays a role in axonemal structure organization and motility	Moore *et al.* (2013)

N.R., no relevant information found in the HPA database; Positive, protein expression was detected in human testis; Negative, protein expression was not detected in human testis; AR, androgen receptor; DMTs, doublet microtubules; GTEx, genotype-tissue expression; HPA, Human Protein Atlas; miRNA, microRNA; N.R., no reported; Uniprot, Universal Protein.

In summary, through integrative cross-species and multi-omics analysis, we identified 38 previously uncharacterized, testis-enriched RBPs with potential roles in male fertility. These genes represent promising candidates for mechanistic investigation and *in vivo* functional validation. Elucidating their roles may uncover novel regulators of spermatogenesis and facilitate the development of clinically relevant gene panels for the diagnosis and personalized treatment of male infertility.

## Clinical potentials of RBPs in male infertility: biomarkers and therapeutic applications

RBPs have emerged as pivotal regulators of spermatogenesis, orchestrating complex transcriptional and post-transcriptional processes essential for male fertility. Their critical roles in germ cell proliferation, differentiation, and maturation underscore their potential as molecular biomarkers and therapeutic targets for male infertility. Advances in genomics, transcriptomics, and RNA-based therapeutics have opened new avenues for leveraging RBPs in clinical practice.

### RBPs as molecular biomarkers for male infertility

The discovery of RBP mutations and dysregulation in male infertility disorders, such as azoospermia, oligozoospermia, teratozoospermia, and asthenozoospermia, highlights their potential as diagnostic biomarkers. The specificity of RBP expression in the testis, coupled with their stage-specific roles in spermatogenesis, makes them ideal candidates for non-invasive diagnostic and prognostic tools.

#### Diagnostic biomarkers in testicular biopsies

RBPs such as DAZL (Dicke *et al.*, 2023), TDRD1 (Gao *et al.*, 2024), MIWI (Deng and Lin, 2002), and YBX2 (Yang *et al.*, 2005) are strongly associated with spermatogenic failure. For instance, decreased or absent expression of DAZL and TDRD1 in testicular tissue biopsies correlates with germ cell loss and meiotic arrest, aiding in the differential diagnosis of NOA.

#### Non-invasive biomarkers in seminal plasma and sperm

Advances in RNA sequencing have enabled the identification of RBP-derived mRNAs and piRNAs in seminal plasma and sperm, reflecting the functional state of spermatogenesis. The detection of aberrant YBX2 transcripts or miRNA-piRNA dysregulation mediated by RBPs in seminal fluid could serve as non-invasive biomarkers for evaluating spermatogenic defects.

#### Prognostic markers for assisted reproductive technologies

RBP profiles in spermatozoa, such as the presence of functional TDRD7 (Tanaka *et al.*, 2011), GRTH (Dufau and Tsai-Morris, 2007), or PRMT5 (Dong *et al.*, 2019), have been linked to successful ICSI outcomes. Tailoring assisted reproductive technologies strategies based on RBP expression patterns may enhance therapeutic success rates in infertile patients.

### Therapeutic potential of RBPs: RBP-targeted drugs and gene editing

Advances in RNA-based therapies and drug discovery have revealed the potential of targeting RBPs to restore spermatogenesis and address infertility caused by RBP dysregulation. These approaches, ranging from RNA-targeted therapeutics to gene editing technologies, offer novel pathways for correcting molecular dysfunctions associated with male infertility.

#### RNA-targeted drugs

RNA-based therapies, including antisense oligonucleotides (ASOs) and siRNAs, enable precise modulation of RBP functions, addressing key molecular pathways disrupted in spermatogenesis.

A prominent example is the use of ASOs to correct aberrant splicing activity in RBPs such as PTBP2 (Dawicki-McKenna *et al.*, 2023), whose dysregulation leads to spermatogenic arrest. Targeted modulation of PTBP2 restores normal splicing patterns, thereby reactivating essential pathways for germ cell differentiation. Similarly, therapeutic modulation of CELF1, an RBP influencing testosterone synthesis during spermatid differentiation (Boulanger *et al.*, 2015), has shown potential to restore hormonal balance and improve differentiation outcomes, emphasizing the importance of RBPs in hormonal regulation and germline development.

RBPs also play a central role in the piRNA pathway, critical for retrotransposon silencing and genomic stability in germ cells. Dysregulation of key RBPs, such as MIWI and HENMT1 (Ding *et al.*, 2019; Li *et al.*, 2024a), compromises chromatin remodeling and leads to spermatogenic defects. Therapeutics that enhance the piRNA pathway could mitigate these disruptions, preventing genomic instability and restoring spermatogenesis.

#### Small molecule inhibitors and stabilizers

Small molecule therapies targeting RBPs have demonstrated their capacity to modulate RNA interactions, stabilize protein complexes, or suppress pathological activity (Jaiswal *et al.*, 2024). These approaches offer unique opportunities for treating infertility by directly addressing molecular dysfunctions in RBPs.

For instance, reverse-turn peptidomimetics targeting SAM68 have shown promise in restoring chromatin regulatory processes critical for germ cell maturation (da Silva *et al.*, 2024). Similarly, compounds such as gossypol and luteolin (Lan *et al.*, 2015; Yi *et al.*, 2018), which disrupt the interaction between MSI1 and its RNA targets, highlight the potential to regulate RNA processing defects associated with spermatogenesis. These small-molecule strategies underscore the versatility of pharmacological interventions in modulating RBP activity for therapeutic benefit.

#### Gene editing approaches

Gene editing technologies, particularly CRISPR-Cas9, have revolutionized the current situation for correcting pathogenic variants in serious diseases, such as sickle cell anemia and β-thalassemia (Frangoul *et al.*, 2021; Sharma *et al.*, 2023). These tools provide potential therapeutic strategy at the genetic level, trying to address the root causes of spermatogenic failure. Gene editing holds promise for correcting mutations in RBPs such as DAZL, TDRD9, and MOV10L1 (Li *et al.*, 2024; Stallmeyer *et al.*, 2024; Wang *et al.*, 2024b), which are essential for germ cell development. Additionally, gene augmentation strategies—such as viral vector-mediated delivery of functional RBP genes—offer an alternative for addressing loss-of-function mutations (Yan *et al.*, 2022), but likewise require careful evaluation before translation into clinical practice. By rectifying these molecular defects, gene editing could potentially restore disrupted spermatogenic processes. However, the application of gene editing to germ cells or embryos remains highly controversial due to ethical concerns and safety risks, including off-target effects and unknown consequences for embryonic development.

#### Emerging drugs and translational applications

Many RBP-targeted drugs currently under investigation for oncology and neurodevelopmental disorders offer significant insights for the development of therapeutics aimed at male infertility (Table 5). For example, VPC-80051, a drug targeting hnRNPA1, restores splicing fidelity, demonstrating its potential to address critical splicing defects in germ cells (Carabet *et al.*, 2019). Similarly, the combination of AIC-47 and imatinib, which modulates splicing and energy metabolism pathways through PTBP1, provides a promising avenue for correcting spermatogenic dysfunction by restoring transcriptomic balance (Shinohara *et al.*, 2016).

**Table 5.** Function and regulatory mechanism of RBP-related drugs.

RBP	Drugs	Functions	Mechanism	In human/animal/cell models	Diseases	Ref.
CIRP	Vaccines containing OVA-CIRP	Generate anti-tumor immunity to enhance immune checkpoint inhibitors efficacy in HCC	Unknown	Intrahepatic tumor mice model, B16-OVA and B16.F10 melanoma cells and B16.HLA-A2	HCC	Silva *et al.* (2020)
CUGBP2	Curcumin	Modulate the expression of CUGBP2 to inhibit pancreatic cancer growth	CUGBP2 inhibits the translation of COX-2 and VEGF mRNA	AsPC-1, MiaPaCa-2, Panc-1, BxPC-3 human and Pan02 mouse pancreatic cancer cells	Pancreatic cancer	Subramaniam *et al.* (2011)
eIF4A	Rocaglamide A	Increase the affinity between eIF4A and RNA, and lead to premature, upstream translation initiation and reduce protein expression	Unknown	HEK 293 Flp-In T-Rex cells	Cancer	Iwasaki *et al.* (2016)
eIF4E	First-in-class small-molecule compound 094	Interact with EIF4E, dissociate RBM38 from EIF4E, and enhance TP53 translation in RBM38- and EIF4E-dependent manners	EIF4E promotes cancer development by preferentially translating oncogenic mRNAs	Cell lines HCT116 and RKO	Cancer	Lucchesi *et al.* (2023)
hnRNPE1	triazole derivative, JL014	Increase the mRNA and protein level of hnRNPE1 to regulate VEC apoptosis and autophagy	HnRNPE1 binds to the promoter and 5′-UTR of *HMBOX1* and active HMBOX1 (a key VEC inhibitor and autophagy inducer) expression	Human umbilical vein vascular endothelial cell	Vascular diseases	Meng *et al.* (2019)
hnRNPH1	MA	Suppress exosome biogenesis and secretion via targeted hnRNPH1	HnRNPH1 mediates Ras-dependent MA suppression	Castration-resistant prostate cancer cells	Prostate Cancer	Datta *et al.* (2017)
hnRNPA1	VPC-80051	Interact directly with hnRNPA1 RBD and reduce AR-V7 messenger levels	HnRNPA1 directly controls the transcription of c-Myc oncoprotein and the production of AR-V7	22Rv1 CRPC cell lines	Prostate Cancer	Carabet *et al.* (2019)
hnRNPA2	Apigenin	No data	HnRNPA2 sensitizes TNBC spheroids to doxorubicin-induced apoptosis and regulates expression of ABCC4 and ABCG2 drug efflux transporters	MDA-MB-231 human TNBC cells	TNBC	Sudhakaran *et al.* (2020)
HuR (ELAVL1)	Dihydrotanshinone-I	Interfere with the association step between HuR and the RNA	HuR orchestrates the stabilization and translation of mRNAs, critical in inflammation and tumor progression, including tumor necrosis factor-alpha	Breast cancer cell lines	Breast cancer	D’Agostino *et al.* (2015)
	Lipoplexes loaded with siRNA silencing HuR expression	Downregulate retinal HuR and VEGF level	Unknown	Streptozotocin-induced diabetic rats	Diabetic retinophathy	Amadio *et al.* (2016)
IMP2 (IGF2BP2)	Benzamidobenzoic acid and ureidothiophene	Through inhibiting IMP2 to suppress tumor growth *in vivo*	Unknown	Zebrafish embryo xenograft model	Colorectal cancer	Dahlem *et al.* (2022)
LIN28	C1632	Increase *let-7* and suppress PD-L1 expression, and lead to reactivation of antitumor immunity *in vivo* and *vitro*	LIN28 inhibits the biogenesis of *let-7*, and promotes PD-L1 expression	Human 293 T, Hela, MCF-7, U2OS, A549, MNNG/HOS, SKLU-1, and mouse TUBO and 4T1 cells	Cancer	Chen *et al.* (2019)
LIN28 and RBFOX1	PROTACs	Mediate selective degradation of LIN28 and RBFOX1 through a cell’s ubiquitination machinery	Unknown	Embryonic NT2/D1 cell line and K562 cells		Ghidini *et al.* (2021)
LIN28B	Epigallocatechin 3-gallate	Inhibit LIN28B/let-7 miRNA interaction, lead to an increase in mature let-7 miRNAs and inhibit NB cell growth	LIN28B regulates the biogenesis of the tumor suppressor let-7 miRNAs	Human NB cell lines	NB	Cocchi *et al.* (2024)
MSI1	Gossypol	Directly interact with MSI1 and inhibit MSI1-RNA interaction	MSI1 acts as a translation activator or repressor of *Numb* and *Apc* mRNA	Colon cancer cell lines, and mouse xenograft model	Colon cancer	Lan *et al.* (2015)
	Luteolin	Inhibit MSI1 binding to RNA and disrupts\cancer phenotypes	MSI1 regulates the expression of stem cell markers and oncogenic factors via mRNA translation/stability	GBM cells	GBM	Yi *et al.* (2018)
MSI2	Largazole	Decrease the protein and mRNA levels of MSI2 and suppress its downstream mammalian target of rapamycin signaling pathway	Unknown	NSCLC and CML cells	NSCLC and CML	Wang *et al.* (2020a)
NCL	Curcumol	Regulate the expression of NCL and influence its function domain	NCL interact with NCL and Akt and regulate cell autophagy	NPC cells	NPC	Liu *et al.* (2023b)
NONO	Auranofin	Inhibiting NONO and suppress glioblastoma multiforme tumor growth	NONO regulates the global intron retention of pre-mRNA and proper splicing of GPX1 and CCN1 pre-mRNAs	An orthotopic xenograft model in mice	Glioblastoma multiforme	Wang *et al.* (2022b)
		Inhibit NONO and suppress cell growth in TNBC	NONO directly interacts with STAT3 protein increasing its stability and transcriptional activity, thus contributing to its oncogenic function	TNBC cell lines	TNBC	Kim *et al.* (2020)
PCBP2	Curcusone C	Inhibit the expression of PCBP2 and induce apoptosis of prostate cancer cells	Unknown	PC-3 cells	Prostate cancer	Huang *et al.* (2023)
PTBP1	AIC-47 in combination with imatinib	Down-regulate the miR-124/PTBP1/PKM2 signaling pathway and strengthen the attack on cancer energy metabolism	PTBP1 functions as AS repressor of PKM1, and results in expression of PKM2	Human leukemia cell lines K562 and KCL-22	Chronic myeloid leukemia	Shinohara *et al.* (2016)
PTBP2	Antisense oligonucleotides	Disrupt PTBP2 binding redirect splicing, increase SYNGAP1 mRNA and protein expression	PTBP2 binds to SYNGAP1 mRNA and promotes AS and nonsense-mediated decay	HEK293T cells, Neuro2A cells, and SH-SY5Y cells	Neurodevelopmental disorders	Dawicki-McKenna *et al.* (2023)
PUM1	Triptolide	Enhance TRAIL sensitivity of pancreatic cancer cells by activating autophagy via downregulation of PUM1	Unknown	Human PC cell lines	PC	Dai *et al.* (2019)
RBM3	Verteporfin	Suppress RBM3-induced proliferation of HCC cells	RBM3 promotes the proliferation of HCC cells through the YAP1 pathway	Human HCC cell lines Huh7, SK-Hep1 and BEL-7404	HCC	Miao and Zhang (2020)
RBM39	Aryl sulphonamides	Promote RBM39 degradation in a DCAF15-dependent manner, which leads to aberrant splicing events and differential gene expression, thereby inhibiting cell cycle progression and causing tumor regression	RBM39 is involved in transcriptional co-regulation and alternative RNA splicing	AML cell lines, murine xenograft models; a murine model of breast cancer, and human NSCLC cell line A549	Acute myeloid leukemia, colorectal cancer, breast cancer and lung cancer	Xu *et al.* (2022c)
	sulfonamides			HCT-116 cells and mouse xenograft models	Cancer	Han *et al.* (2017)
	Indisulam (E7070)			Human T-ALL cell lines	T-cell acute lymphoblastic leukemia	Ji *et al.* (2024)
SAM68	Reverse-turn peptidomimetic drugs	Selectively eliminate CSC activity upon engaging Sam68	SAM68 contributes to chromatin regulation processes	CSC	Human tumors	da Silva *et al.* (2024)
SMN	Nusinersen	Modify pre-messenger RNA splicing of the SMN2 gene and thus promotes increased production of full-length SMN protein	Unknown	A randomized, double-blind, sham-controlled, phase 3 efficacy and safety trial of nusinersen in infants with SMA	SMA	Finkel *et al.* (2017), Gowda *et al.* (2023)
THOC1	Andrographolide	Reduce the expression and impair homeostasis of Thoc1, suppress cancer stem cells properties, and delay tumor growth	THOC1 regulates the elongation, processing and nuclear export of mRNA	4T1-implanted orthotopic mouse model	TNBCs	Chou *et al.* (2022)

CML, chronic myeloid leukemia; CSC, cancer stem cells; GBM, glioblastoma; HCC, hepatocellular carcinoma; MA, Manumycin-A; NB, neuroblastoma; NPC, nasopharyngeal carcinoma; NSCLC, non-small cell lung cancer; PC, pancreatic cancer; RBP, RNA-binding protein; SMA, spinal muscular atrophy; TNBC, triple-negative breast cancer; VEC, vascular endothelial cell.

Emerging therapies targeting CELF1 have also shown promise in regulating testosterone synthesis and differentiation processes in spermatids. These approaches not only address hormonal imbalances but also improve germ cell development, positioning CELF1 modulation as a novel strategy for infertility treatment. In addition, drugs targeting SAM68 and MSI1 focus on restoring RNA processing and chromatin interactions, which are crucial for germ cell maturation and overall spermatogenic integrity. These examples underscore the broad potential of RBP-targeted therapeutics to modulate pathways integral to spermatogenesis. By building on these foundational mechanisms, these drugs open new avenues for innovative treatments to address male infertility.

## Conclusions, outstanding questions, and future perspectives

RBPs are indispensable for transcriptional and post-transcriptional processes during spermatogenesis, orchestrating germ cell proliferation, differentiation, and maturation. This review systematically analyzes genetic variants, known genes, functional roles, uncharacterized candidates, and clinical potentials of RBPs in male infertility. Leveraging state-of-the-art datasets and experimental insights, it examines pathogenic variants and VUS, and elucidates the GDRs. Furthermore, it explores known RBP functions across spermatogenesis stages and identifies candidate RBP genes. By integrating these findings, this work provides a comprehensive framework to advance the genetic understanding of RBPs, and their potential as clinical biomarkers and therapeutic targets in male infertility.

Firstly, through a comprehensive analysis of the ClinVar and PubMed databases, this work categorizes all male infertility-related RBP gene variants into 177 pathogenic variants in 62 RBP genes and 91 VUS in 35 RBP genes. This stratification not only elucidates the known genetic landscape but also highlights VUS as a fertile ground for future research into their pathogenic potential. Notably, 15 RBPs were supported by moderate to definitive clinical evidence of association with male infertility, according to the IMIGC database. These findings offer a high-confidence foundation for diagnostic gene panel development.

Secondly, we analyze the fertility phenotypes and functional roles of 1744 mouse testicular RBP genes, 124 of which harboring knockout mouse models with male infertility. This review systematically examines their stage-specific regulatory mechanisms during spermatocytogenesis, spermatidogenesis, and spermiogenesis. These insights bridge the gap between functional studies in animal models and translational research in humans, offering mechanistic insights into male infertility phenotypes.

Thirdly, by searching human homologous RBP genes to 408 mouse testis-enriched RBP genes, 163 human testis-enriched RBP genes were found. Among them, 38 lacking knockout mouse models were screened as candidate RBP genes. These uncharacterized genes represent a promising pool for future investigations in male infertility.

Fourthly, the review also explores the clinical relevance of RBPs, emphasizing their potential as biomarkers for male infertility and as therapeutic targets. It highlights RNA-targeted drugs, small molecules, and gene editing technologies as promising strategies for treating RBP-related spermatogenic dysfunction.

Therefore, this comprehensive effort provides a significant foundation for understanding the role of RBPs in male infertility, integrating genomic, transcriptomic, and functional data to identify both established and candidate regulatory pathways.

We really appreciate the pioneering efforts of researchers who have significantly advanced the study of RBPs in spermatogenesis and male infertility. Their innovative explorations, particularly in identifying and characterizing RBP functions in germ cell development (as summarized in this review), have laid the groundwork for understanding the intricate molecular mechanisms driving male reproduction.

We are also particularly grateful for the seminal work by Li *et al.*, *The landscape of RNA binding proteins in mammalian spermatogenesis* (Li *et al.*, 2024c), which systematically charted the RBP atlas across germ cell developmental stages. Their comprehensive dataset has served as a crucial foundation for this review, enabling the integration of large-scale RBP profiling with functional and clinical data. The foundational studies by these experts have not only elucidated critical regulatory pathways but also inspired the next generation of reproductive biologists. Their contributions have been instrumental in enabling us to prepare this article, which we hope will serve as a valuable resource for those seeking to further unravel the complexities of RBPs in reproductive biology and clinical applications.

Future studies are needed to answer the following questions:

Functional validation: What specific roles do the 38 candidate RBPs play in spermatogenesis? Are knockout or knock-in mouse models available to confirm their functions? If not, what approaches can be used to systematically evaluate their roles?

Gene reclassification: Among the genes listed in the IMIGC database, only 15 RBP genes are confidently linked to male infertility. How should the remaining RBP genes, currently supported by limited or no evidence, be systematically reclassified?

Reassessment of VUS: This study identified 91 VUS in 35 RBP genes. How many of these variants show potential pathogenicity? What functional tools or models are needed to enable their definitive classification?

Environmental and epigenetic effects: Do environmental factors such as toxins or endocrine disruptors affect RBP expression or function in germ cells? Can these changes lead to epigenetic modifications, and are such effects reversible?

Therapeutic delivery and safety: How can RNA-targeted therapies or gene editing tools for RBP-related infertility be delivered effectively and safely to testicular tissue? What are the long-term risks for the health of offspring?

Answers to these questions, will be beneficial to interpret the roles of RBPs in spermatogenesis, but may also be helpful to diagnose and treat cases of relevant male infertility.

## Data Availability

The data supporting the findings of this review are derived from publicly available sources, including peer-reviewed publications and genomic databases. Any additional information or unpublished data referenced in the manuscript can be provided upon reasonable request to the corresponding author.
